# Performance of Cu/ZnO Nanosheets on Electrospun Al_2_O_3_ Nanofibers in CO_2_ Catalytic Hydrogenation to Methanol and Dimethyl Ether

**DOI:** 10.3390/nano13040635

**Published:** 2023-02-05

**Authors:** Itzhak I. Maor, Svetlana Heyte, Oren Elishav, Meirav Mann-Lahav, Joelle Thuriot-Roukos, Sébastien Paul, Gideon S. Grader

**Affiliations:** 1 The Wolfson Department of Chemical Engineering, Technion—Israel Institute of Technology, Haifa 3200003, Israel; 2 Université de Lille, Centre National de la Recherche Scientifique (CNRS), Centrale Lille, Université d’Artois, UMR 8181, Unité de Catalyse et Chimie du Solide (UCCS), F-59000 Lille, France; 3 The Nancy & Stephan Grand Technion Energy Program (GTEP), Technion—Israel Institute of Technology, Haifa 3200003, Israel

**Keywords:** electrospinning, nanofibers, catalyst, hydrogenation, CO_2_, methanol, dimethyl ether (DME)

## Abstract

The synthesis of methanol and dimethyl ether (DME) from carbon dioxide (CO_2_) and green hydrogen (H_2_) offers a sustainable pathway to convert CO_2_ emissions into value-added products. This heterogeneous catalytic reaction often uses copper (Cu) catalysts due to their low cost compared with their noble metal analogs. Nevertheless, improving the activity and selectivity of these Cu catalysts for these products is highly desirable. In the present study, a new architecture of Cu- and Cu/Zn-based catalysts supported on electrospun alumina nanofibers were synthesized. The catalysts were tested under various reaction conditions using high-throughput equipment to highlight the role of the hierarchical fibrous structure on the reaction activity and selectivity. The Cu or Cu/ZnO formed a unique structure of nanosheets, covering the alumina fiber surface. This exceptional morphology provides a large surface area, up to ~300 m^2^/g, accessible for reaction. Maximal production of methanol (~1106 g_methanol_Kg_Cu_^−1^∙h^−1^) and DME (760 g_DME_Kg_Cu_^−1^∙h^−1^) were obtained for catalysts containing 7% wt. Cu/Zn with a weight ratio of 2.3 Zn to Cu (at 300 °C, 50 bar). The promising results in CO_2_ hydrogenation to methanol and DME obtained here point out the significant advantage of nanofiber-based catalysts in heterogeneous catalysis.

## 1. Introduction

Over the last few decades, the CO_2_ concentration in the atmosphere has increased rapidly, reaching an unprecedented level of 415 ppm, predominantly due to fossil fuel burning. The rising levels of CO_2_ and other greenhouse gasses cause global warming climate change [1,2,3]. This problem can be mitigated by trapping CO_2_ either from the atmosphere or from emission sources and converting it into value-added chemicals, such as light olefins, hydrocarbons, alcohols, and, specifically, methanol (MeOH) and dimethyl ether (DME) [4,5,6,7,8]. Methanol is a vital industrial chemical that serves as a solvent in many processes and as a raw material for other chemicals, such as formaldehyde and acetic acid. Furthermore, MeOH and DME can serve as alternative energy carriers as multi-purpose fuels. Methanol can be used as a precursor to the familiar gasoline in the methanol-to-gasoline process [9,10,11,12].

At present, industrial-scale methanol synthesis is carried out almost exclusively using the catalytic reaction of synthesis gas (syngas) over copper, zinc-oxide, and aluminum-oxide composite (Cu\ZnO\Al_2_O_3_) catalysts [9,11]. Overall, syngas (mixture of CO, CO_2_, and H_2_ gases) reacts by the following reversible reactions (1,2) to form methanol [11]:

(1) $$\mathrm{CO}+2H_{2}↔{\mathrm{CH}}_{3}\mathrm{OH}\;\;\;\;\;\Delta H_{T=300K}=-90.77_{\frac{\mathrm{KJ}}{\mathrm{mol}}}$$

(2) $${\mathrm{CO}}_{2}+3H_{2}↔{\mathrm{CH}}_{3}\mathrm{OH}\;+H_{2}O\;\;\;\;\;\Delta H_{T=300K}=-49.16_{\frac{\mathrm{KJ}}{\mathrm{mol}}}$$

The mechanism of methanol formation from syngas is still under investigation. However, it is widely accepted that metallic Cu is the active phase in methanol synthesis via either CO or CO_2_ hydrogenation [11,13]. Therefore, in recent years, many studies investigated pure CO_2_ hydrogenation to methanol over Cu-based catalysts [1,4,10,14,15]. Unfortunately, a major drawback of this approach is the reverse water gas shift (RWGS) reaction (3), which competes with reaction (2) and is also catalyzed by the Cu-based catalysts [11,16].

(3) $${\mathrm{CO}}_{2}+H_{2}↔\mathrm{CO}+H_{2}O\;\;\;\;\;\Delta H_{T=300K}=41.21_{\frac{\mathrm{KJ}}{\mathrm{mol}}}$$

To reduce the extent of the RWGS reaction and optimize the catalyst’s effectiveness towards methanol formation (i.e., methanol selectivity, catalytic activity, and stability), in many cases, Cu is supported by other metallic/ceramic materials, mainly ZnO and Al_2_O_3_, as in commercial catalysts [17,18,19,20,21,22,23,24,25,26,27,28,29]. While ZnO was shown as taking an active part in reaction promotion (sometimes referred as cocatalyst) [18,20,21,27,28,29,30], Al_2_O_3_ is considered a chemically inert component that serves mainly as a stabilizer of Cu/ZnO particles dispersed in the catalyst [11,31]. Still, in few works, where Al_2_O_3_ concentration in the catalysts was relatively high, Brønsted and Lewis acid sites on its surface served as an effective catalyst for MeOH dehydration to dimethyl ether (DME) (4) [23,31,32,33]. Since DME is also a valuable feedstock and clean energy source [34], its production by the secondary reaction from MeOH is highly desirable in many works [34,35,36].

(4) $$2{\mathrm{CH}}_{3}{\mathrm{HO}}_{\left(g\right)}↔{\mathrm{CH}}_{3}{\mathrm{OCH}}_{3}{}_{\left(g\right)}+H_{2}O_{\left(l\right)}\;\;\;\;\;\Delta H_{R}^{0}=-23.50_{\frac{\mathrm{KJ}}{\mathrm{mol}}}$$

Achieving simultaneously high methanol and DME selectivity and CO_2_ conversion ($X_{CO_{2}}$) in Cu-based catalysts is challenging [1]. For example, Yao et al. [37] achieved high methanol selectivity of 80%, but with low CO_2_ conversion of approximately 1.5% (*Y_MeOH_* = 1.2%) using Cu-In-Zr-O catalysts (at 523 K and 2.5 MPa). On the other hand, Li et al. [24] achieved higher CO_2_ conversion of ~17% but lower selectivity of ~45% (*Y_MeOH_* = 7.65%) at similar reaction conditions (533 K and 3 MPa) using Cu-Al-Ce-O catalysts. Additionally, in the same work, the trend of increasing conversion with decreasing methanol selectivity as a function of reaction temperature (constant pressure) is presented. At 473 K, selectivity of 85% and conversion of 2.9% were obtained, while at 553 K, the selectivity decreased to 22% and conversion increased to 23.7%. The methanol yield in these works was 2.5% and 5.2%, respectively. A similar trend was presented by Lam et al. [31], varying residence time in the reactor (instead of temperature). Working with γ-Al_2_O_3_-Cu catalyst for methanol and DME production (at 503 K and 2.5 MPa), the authors obtained $X_{CO_{2}}$ = 0.9%, *S_MeOH_* = ~16.5%, and *S_DME_* = ~27.5% at contact time of 0.15 s∙g_cat_∙mL^−1^, but $X_{CO_{2}}$ = 5.7%, *S_MeOH_* = ~9%, and *S_DME_* = ~14% at contact time of 2.5 s∙g_cat_∙mL^−1^.

In other cases, where both conversion and selectivity are relatively high, reaction conditions are extreme or platinum-based catalysts were used. For instance, Samson et al. [38], using Cu-Zr-O catalysts and applying a pressure of 80 bar and a temperature of 533 K in the reactor, managed to obtain relatively high conversion and selectivity at the same time ($X_{CO_{2}}$ = 15%, *S_MeOH_* = 86%, and *Y_MeOH_* = 12.9%). On the other hand, Men et al. [39] conducted the reaction under relatively low temperature (303 K) and under atmospheric pressure to achieve quite impressive results ($X_{CO_{2}}$ = 37%, *S_MeOH_* = 62.6%, and *Y_MeOH_* = 23%); however, the authors used an expensive Pt-In-O-based catalyst and a special dielectric barrier discharge (DBD) plasma reactor.

In addition to the catalysts’ composition and reaction conditions, the catalytic performance is greatly affected by the catalyst’s structure. [16] Le Valent et al. [28] and Tisseraud et al. [27,29] investigated the synergistic effect of Cu-ZnO nanoparticle catalysts in methanol synthesis. Their main objective was to increase methanol selectivity over the RWGS reaction. It was shown that Cu@ZnO core–shell catalysts were superior in their activity and selectivity towards methanol formation ($X_{CO_{2}}$= 2.3%, *S_MeOH_* = 100%, and *Y_MeOH_* = 2.3%), in comparison with Cu-ZnO catalysts produced by coprecipitation or by mechanical mixing ($X_{CO_{2}}$= ~0.9%, *S_MeOH_* = 100%, and *Y_MeOH_* = 0.9%). All catalytic measurements were conducted at 523 K and 30 bar. This feature was attributed to a greater contact areas between Cu and ZnO in the Cu@ZnO core–shell catalysts, in which a Cu_x_Zn_(1−x)_O_y_ composite oxide phase with oxygen vacancies is formed and serves as the active site for methanol formation. According to a simplified mechanism proposed in these works, hydrogen is adsorbed and dissociated on the Cu surface. Adsorbed hydrogen atoms directed, via spillover, both to ZnO surface and ZnO_x_ at the Cu/ZnO interface that serve as adsorption sites for CO_2_. It was then shown that hydrogenation of CO_2_ on the ZnO surface is more efficient for CO formation via RWGS, while ZnO_x_ is the active site for methanol formation.

The most commonly used catalysts in this field are nanoparticle-based powders [10]. However, other catalysts’ morphologies are expected to improve performance [1,10]. One-dimensional nanostructures, such as nanofibers and nanobelts, could be of a great advantage in such a heterogeneous reaction. In fact, nanofibers possess high surface-area-to-volume ratio beneficial for heterogeneous catalysis [40,41,42]. Furthermore, novel porous or core–shell shapes with designed architecture and composition are applicable to nanofibers and can be ideal for catalytic purposes [43,44,45]. Due to their structure, nanofibers are expected to exhibit enhanced stability under stream compared with the powder nanoparticle-based catalysts [46,47,48].

To maximize the benefits of the above qualities, Sun et al. [49] produced catalysts based on carbon nanotubes as a support on which a Cu\ZrO_2_ catalytic layer was anchored. This catalyst showed improved performance ($X_{CO_{2}}$ = 11.5%, *S_MeOH_* = 75%, *Y_MeOH_* = 8.64%, and methanol production of 1022.1 g_MeOH_kg_Cu_^−1^h^−1^ at 533 K and 3 MPa). In addition, it was shown that the fibrous structure provided the catalyst with greater stability under reaction conditions.

In the present study, we propose a different approach to prepare such nanofibrous catalysts by utilizing electrospinning (ES), which produces 1D nanostructures with controlled morphology and phases [41,42,50]. These resulting nanofibers demonstrate a unique surface architecture with high BET surface area and meso- and macro-porous surface. Nanofibrous catalysts were produced by decoration of electrospun alumina nanofibers (AlNFs) with Cu or Cu&Zn, to give rise to high conversion and methanol production, beyond the current state of the production in a hierarchical fibers’ structure of nanosheets anchored to the alumina surface. The exceptional surface structure of produced catalysts provides large accessible surface for reaction, which is expected. To the best of our knowledge, this is the first time that this type of hierarchically structured catalyst is used for the hydrogenation of CO_2_ into high value-added chemicals such as methanol and DME. We have investigated the interplay between the nanofibers’ composition, morphology, and catalyst performance. Up to 1106 g_MeOH_Kg_Cu_^−1^∙h^−1^ and 760 g_DME_Kg_Cu_^−1^∙h^−1^ were obtained at 50 bar and 300 °C with AlNFs-1Cu2.3Zn-7 catalyst containing 7% wt. of Cu&Zn. Obtained results demonstrate the beneficial effect of surface structure of decorated electrospun alumina nanofibers that provides large accessible surface for reaction in comparison with the other Al_2_O_3_-ZnO-Cu-based catalysts (see Table S1). The promising results obtained in this work open the way for further optimization of this kind of catalyst and increase their performance in methanol and DME syntheses.

## 2. Experimental Section

### 2.1. Nanofibers Catalysts Preparation

Electrospun alumina nanofibers AlNFs were produced similarly to earlier work [43]. The ES precursor solution consisted of 13 wt% Al(acetylacetonate)_3_ (Aldrich, St. Louis, MO, USA), which was mixed and stirred with acetic acid glacial (Frutarom, Herzliya, Israel) (60 wt%) and 96% ethanol (Bio-Lab, Jerusalem, Israel) (20 wt%) for 30 min until a homogenous yellowish solution was obtained. Polyvinylpyrrolidone (PVP) (*M_w_* = 1,300,000 g/mol, Aldrich, St. Louis, MO, USA) was added to the solution (7 wt%). After overnight stirring, the final viscosity of the precursor solution was ~300 cPs.

The precursor solution was electrospun in the NS 24 Electrospinning Machine (Inovenso, Turkey). Applied voltage was 16kV/−3kV with a tip-to-collector distance (TCD) of 15 cm and a precursor feed rate of 1 mL/h. The relative humidity inside the system container was 45%, and the temperature was in the range of 20–30 °C. After ES, the resulting “green” fibers mats were dried overnight under vacuum at 40 °C. Next, the fibers mats were cut to approximately 2 × 2 cm^2^ squares and calcined in air at 973 K for 1 h in a box furnace (Nabertherm, Germany), as described elsewhere [43].

After calcination, the resulting alumina nanofibers were decorated with Cu and Zn precursors by impregnation of aqueous solutions containing Cu(NO_3_)_2_ (Carlo Erba Reagents S.r.l., Cornaredo, Italy), Zn(NO_3_)_2_ (Alfa Aesar, Haverhill, MA, USA), or Cu(NO_3_)_2_+Zn(NO_3_)_2_, keeping a constant Zn/Cu wt. ratio of 2.33, as this ratio showed high yields to methanol in previous works [27,28]. The total metal loading defined as $\frac{mass\;of\;Cu\;or\;Zn\;or\;Cu+Zn}{mass\;of\;AlNFs}\cdot 100\;$, was changed from 1.5% to 10% (1.5, 3, 7, and 10%). Next, the samples were placed in a preheated furnace at 80 °C and dried for 24 h. Finally, dried product was heated in air at 400 °C for 6 h (heating rate 5 °C min^−1^) to decompose nitrate residues and form metal–oxide phase. In total, 12 catalysts were synthetized (Table S2).

### 2.2. Materials Characterization

Raw, sintered, and decorated alumina nanofibers were characterized using a high-resolution scanning electron microscope—HRSEM (ULTRA plus; Zeiss, Switzerland). Elemental distribution in decorated alumina nanofibers samples was performed qualitatively using mapping function of the HRSEM’s EDS detector (XFlash Detector 4030, Bruker AXS, Billerica, MA, USA).

Quantitative analysis of Cu and Zn in the decorated alumina nanofibers was carried out by inductively coupled plasma–optical emission spectroscopy (ICP-OES) (720-ES ICP-OES, Agilent, Santa Clara, CA, USA) with axially viewing and simultaneous CCD detection. Data were treated in the ICP ExpertTM software (version 2.0.4) to allow estimations of the weight percentages of elements in each sample. To achieve representative results, triplicates of each sample were prepared for analysis, and each replicate was analysed three times.

Elemental concentrations in the samples were also determined by energy dispersive X-ray fluorescence (EDXRF) analysis (M4 Tornado, Bruker, Billerica, MA, USA). A Rhodium X-ray tube (50 kV/600 μA (30 W)) with a polycapillary lens was used, enabling excitation of an area of 200 μm. The detector used for these measurements was a Silicon-Drift-Detector Si(Li) with <145 eV resolution at 100,000 cps (Mn Kα), which was cooled with a Peltier cooling (253 K). The measurement was taken under a vacuum (20 mbar). Quantitative analysis was conducted using fundamental parameter (FP). More than 30 points were measured for each sample to cover the entire surface sample.

Specific surface area (SSA) and pore structural properties of support/scaffold and decorated alumina nanofibers were investigated by BET analysis at 77 K (3Flex apparatus, Micromeritics, USA). Phase composition of decorated and not decorated alumina nanofibers were characterized by X-ray diffraction—XRD (SmartLab 9 kW; Rigaku, Japan). Average crystal sizes of decorated and undecorated samples were determined using the Scherrer equation.

High-resolution transmission electron microscopy (HRTEM) imaging was carried out using a ThermoFischer Titan Themis 300 S/TEM with a Schottky X-FEG electron source operating at 300 kV. Particles of the catalyst were deposited on a holey carbon film supported on a gold grid without using solvents. The microscope is equipped with a probe aberration corrector and a monochromator, allowing a special resolution of 70 pm and an energy resolution of 150 meV. The microscope is equipped with a Super-X windowless 4 quadrant SDD (silicon drift detector) detection system for the STEM−energy-dispersive X-ray (EDX) mapping and several annual dark field detectors. The experiment was performed with a spot size of approximately 500 pm, a semiconvergence angle of 20 mrad, and a probe current lower than 100 pA. For the high-angle annular dark-field (HAADF) images, the collection angles were between 50 and 200 mrad. The STEM−EDX mapping was performed with a dwell time of 15 μm/px, with continuous scanning over several frames during a total time of 10–15 min per acquisition. 

### 2.3. Catalytic Testing

The catalytic performance was evaluated at the REALCAT platform (Villeneuve d’Ascq, France) using a Flowrence high-throughput unit (Avantium, Netherlands), which comprises 16 isothermal fixed-bed, stainless steel micro-flow bed reactors, and has the capability to simultaneously screen 16 catalysts (see Figure S1). Each catalyst was loaded into a stainless steel fixed-bed reactor after mixing it with a silicon carbide (SiC) diluent (100 μm) at a weight ratio of 1:1. Prior to test, the catalysts were activated (in situ) by reduction of CuO to Cu metal under 3 mL/min of a mixture of H_2_ and N_2_ (H_2_ /N_2_ = 1/5 vol.), at a pressure of 1 bar and a temperature of 300 °C (heating rate 1 °C/min), for 3 h. After reduction, the system was cooled to 150 °C and purged with the reaction mixture gas (feed) until stabilization. The inlet gaseous mixture (in mol%) was composed of 77.5% of H_2_, 19.4% of CO_2_, and 3.1% of He (used as internal standard for GC analysis), corresponding to a H_2_/CO_2_ molar ratio of 4:1. Feed flowrate of each of the 16 reactors was set to 1.3 mL/min (STP), and each reactor was loaded with 30 mg of catalyst so that the weight hourly space velocity of CO_2_ (WHSV) was set at 1 hr^−1^.

Catalysts were tested (screened) at 10, 30, and 50 bar and at 225, 250, 275, and 300 °C. The screening procedure was performed as follows: First, the pressure was increased to 10 bar. Then, the temperature was increased by 1 °C/min to 225 °C, which is the first temperature tested. After a stabilization time of one hour, the effluent of each reactor was analysed by GC, and the temperature was increased by 25 °C to reach the next tested temperature, namely 250 °C. At 250 °C the same one-hour stabilization time and outgas analysis was performed. This procedure was repeated for 275 and 300 °C. The entire process was then carried out at pressures of 30 and 50 bar. The durations of the catalytic experiments at 225 °C at each pressure was 7.5 h and 12.5 h at 250–300 °C.

The reaction products were analyzed using an on-line GC (Agilent Technologies, model 7890A, United States) equipped with two TCD (PPQ and HayeSepQ/Molecular Sieve columns) and an FID (CP-Sil5 column) detectors. The catalysts’ performance was characterized by CO_2_ conversion, yield, and selectivity to CO, CH_4_, methanol, and dimethyl ether (DME). Details of the calculations of conversion, selectivity, yield, and thermodynamic limit are presented in Supplementary Materials.

## 3. Results and Discussion

### 3.1. Nanofiber Characterization

**Figure 1:**
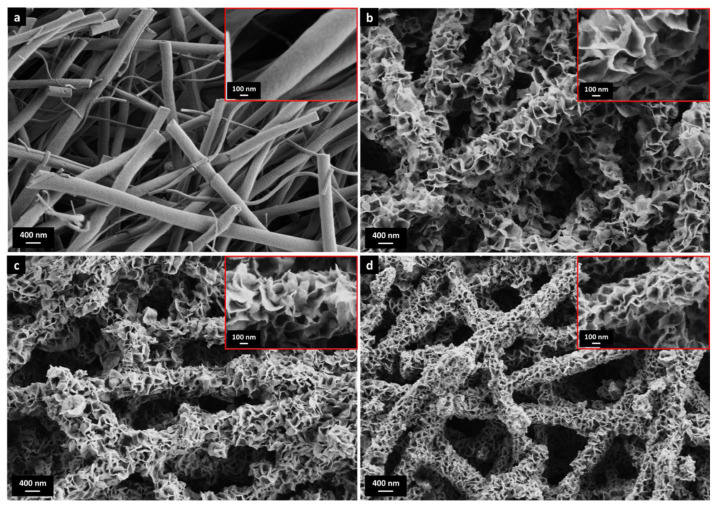
HRSEM images of (**a**) AlNFs and (**b**–**d**) AlNFs decorated by 7 wt% Zn in the form of ZnO (sample AlNFs-Zn-7), 7 wt% Cu in the form of CuO (sample AlNFs-Cu-7), and 7 wt% Zn&Cu in the form of ZnO&CuO (AlNFs-1Cu2.3Zn-7) in a wt. ratio of 2.3 Zn/Cu. The inserts are larger magnifications.

Raw and calcined nanofibers (AlNFs) had a round, smooth surface (Figure 1a, Figures S2 and S3) and a bimodal diameter distribution, with average diameters of 540 nm and 240 nm, respectively (Figure S4). The nanofibers decorated by ZnO, CuO, or ZnO&CuO were termed AlNFs-Zn, AlNFs-Cu, and AlNFs-1Cu2.3Zn. As shown in Figure 1b–d, for 7% wt. loading, the fiber surface is covered with nanosheets (<10 nm thick) extending perpendicularly from the fiber surface. This unique structure was observed at all loadings (1.5, 3, 7, and 10% wt. of Zn, Cu, or Zn&Cu). However, as expected, nanosheets are larger at higher metal–oxide loadings. Representative HRSEM images of AlNFs-1Cu2.3Zn at different loadings are presented in Figure S5.

Nitrogen physisorption performed on initial AlNFs support showed a type-IV isotherm with hysteresis type 2 (Figure S6a) due to the fibers’ meso-porosity [51]. This was also consistent with the sample’s BJH desorption average pore diameter of 4.1 nm and BET surface area of 257 m^2^/g [51]. On the other hand, decorated AlNFs showed type-II isotherm with hysteresis type 3 (Figure S6b–g), corresponding to the presence of elongated, slit-like macropores in the sample [51], consistent with the relatively large gaps between the nanosheets observed in Figure 1 and Figure S5. Compared with the initial AlNFs support, the BET surface area of decorated fibers initially tended to increase at low amounts of metal–oxide loading and then decreased as the amount of loading increases (Table S2). We believe that at low loading, some of the support’s mesopores were still exposed, while at higher loadings they gradually became blocked; thus, the specific surface area (SSA) decreased.

Similar nanosheet-covered ceramics have been reported for various metal oxides in different applications. For example, Tian et al. produced microparticle pigment of Bi_2_W_1−x_Mo_x_O_6_ [52], Lu et al. produced nanocomposites photocatalyst of SnO_2_ [53], Li et al. produced thermal insulators microspheres of Fe-doped Si–C–N [54], and Landman et al. produced Ni(OH)_2_ nanosheets on metallic Ni-nanofibers in an electrochemical process where the hydroxide layer grew via an electro-oxidation process [55]. In all mentioned works, there is no explicit explanation of the formation of such a structure; however, this appears to be a dissolution/precipitation process during synthesis. In our case, the structure is formed during drying, after impregnation (Figure S7), where precipitation takes place.

Elemental analyses using ICP and XRF were performed to confirm the amount of each element in the samples (Table S3). Results of both techniques werre relatively close to the expected values, indicating that the amount of Cu and Zn in the deposit was identical to the starting solution. Additionally, SEM-EDS was performed on representative samples, including AlNFs decorated with 7% Cu, 7% Zn, and 7% Zn&Cu, to ensure homogeneous distribution of the elements in the fibers’ mat (Figure S8). The measurements showed homogeneously distributed elements in the fibers’ mat.

**Figure 2:**
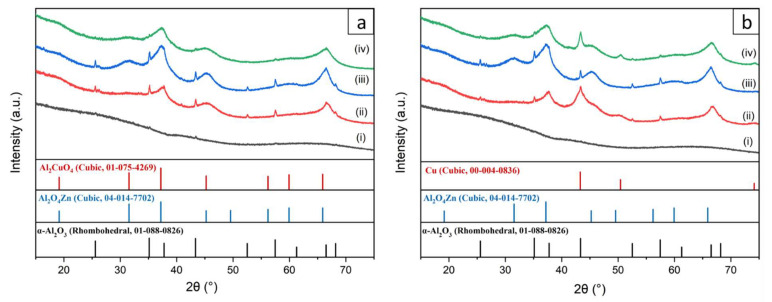
XRD patterns of (**a**) fresh and (**b**) reduced samples. (i)—(gray curve) AlNFs, (ii)—(red curve) AlNFs decorated by 10 wt% Cu (catalyst AlNFs-10-Cu), (iii)—(blue curve) Al-O NFs decorated by 10 wt% Zn (catalyst AlNFs-10-Zn), and (iv)—(green curve) AlNFs decorated by 10 wt% Zn&Cu in a wt. ratio of 2.3 Zn/Cu (catalyst AlNFs-10-1Cu2.3Zn).

The crystallographic structures of the initial and coated fibers with 10 wt% Cu, 10 wt% Zn, and 10 wt% Zn&Cu in their fresh and reduced form are shown in Figure 2. AlNFs presented an amorphous alumina phase with small peaks corresponding to α-Al_2_O_3_ (corundum) phase. It is known that α-Al_2_O_3_ forms at much higher temperatures (~1100 °C) [56] than what is used in this study to form Al-O-NFs (700 °C). The formation of α-Al_2_O_3_ is attributed to local overheating in the fibers, resulting from the exothermic thermal decomposition of their organic content during calcination. After calcination, decorated samples presented much clearer peaks of α-Al_2_O_3_. This observation is surprising since during calcination, the decorated samples were heated only up to 400 °C. In addition, thermal decomposition of metal-NO_3_ precursors is not necessarily exothermic [57]; therefore, the sample’s temperature was not expected to rise above the set-point temperature in the furnace. Here, growth of the α-Al_2_O_3_ grains was promoted by the presence of either ZnO and/or CuO on the α-Al_2_O_3_ grains [58,59]. This phenomenon was seen previously when Al_2_O_3_ was interacting with other oxides, such as CaO and SiO_2_ [60,61].

Apart from distinct alumina peaks, the diffraction of the fresh samples showed some broad characteristic peaks which, in the case of copper loading, matched the Al_2_CuO_4_ spinel and, in the case of the zinc loading, matched the zinc aluminum oxide gahnite (Al_2_O_4_Zn). After reduction in H_2_/N_2_ atmosphere, the diffraction pattern did not change in the cases of alumina and ZnO, but in the case of alumina and Cu loaded catalysts, it showed a transition from the CuO (Al_2_CuO_4_) phase to metallic copper. On the basis of the Scherrer equation, the broad peaks of AlNFs decorated with 10 wt% Cu and 10 wt% Zn represented crystallites size in the range of 1.6–4.2 nm. In contrast, the distinct α-alumina peaks in the samples were correlated to much larger crystallites of ~50 nm (Table S4).

**Figure 3:**
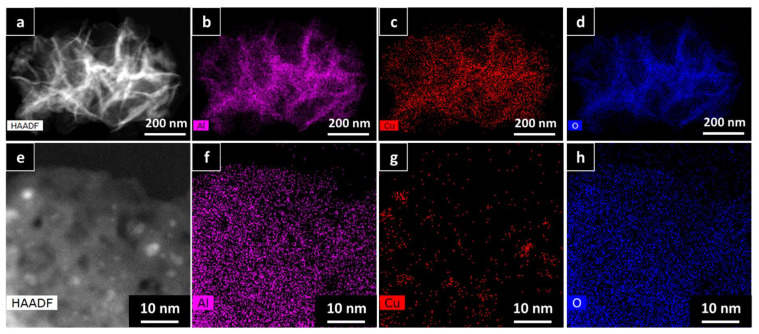
HAADF-HRSTEM EDX patterns of AlNFs decorated with 10 wt% of Cu (AlNFs-Cu-10 catalyst) reduced prior to the analysis: (**a**–**d**) small magnification and (**e**–**h**) large magnification.

**Figure 4:**
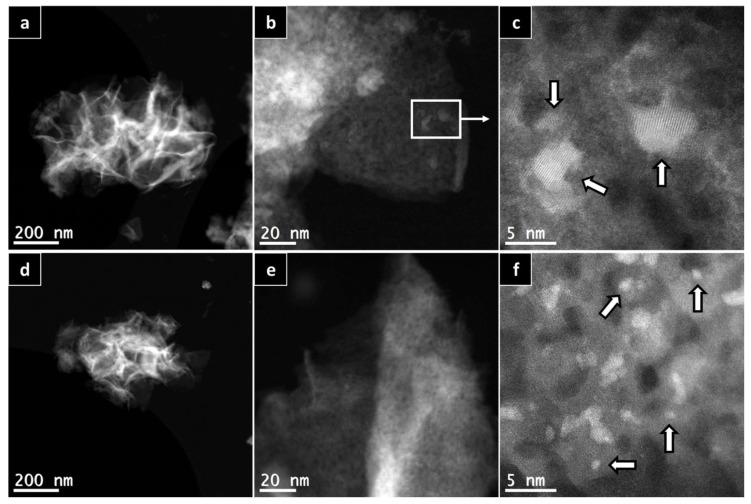
HAADF-HRSTEM patterns of AlNFs decorated with 10 wt% of Cu (**a**–**c**) and AlNFs decorated with 7 wt% of Cu (**d**–**f**). Catalysts were reduced prior to the analysis. White arrows point to few Cu nanoparticles in the sample.

To further prove the calculations using the Scherrer equation, HAADF-HRSTEM imaging and EDX were utilized on the AlNFs decorated with 10 wt% of Cu (AlNFs-Cu-10 catalyst) reduced prior to the analysis (Figure 3). In a small magnification, it was observed that elements were homogenously distributed in the sample (Figure 3a–d); however, in larger magnification, small (~1–5 nm) Cu islands were observed (Figure 3a–d and Figure 4a–c), consistent with the XRD results. Moreover, HAADF-HRSTEM imaging conducted on AlNFs decorated with 7 wt% of Cu (AlNFs-Cu-7 catalyst) reduced prior to the analysis (Figure 4d–f). Compared with the AlNFs-Cu-10, the Cu particles in AlNFs-Cu-7 were better dispersed and had smaller size (<1 nm), pointing to their potentially improved catalytic activity.

### 3.2. Catalytic Performance

**Figure 5:**
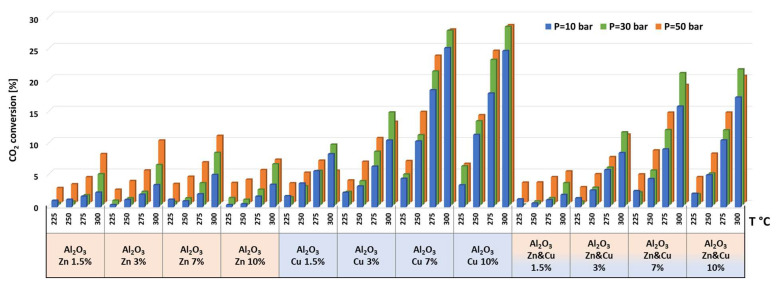
CO_2_ conversions for all catalysts at pressures of 10, 30, and 50 bar and temperatures of 225, 250, 275, and 300 °C.

**Figure 6:**
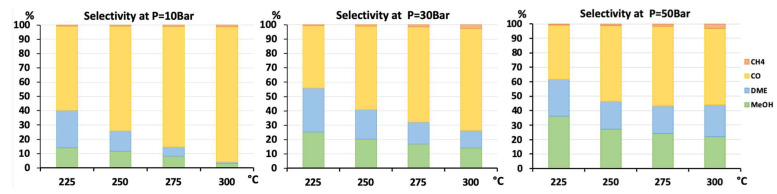
Selectivity to CH_4_, CO, DME, and methanol for AlNFs-Cu catalyst containing 10wt.% of Cu at pressures of 10, 30, and 50 bar and temperatures of 225, 250, 275, and 300 °C.

**Figure 7:**
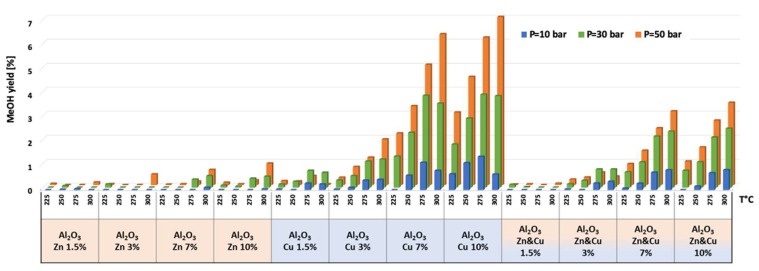
Methanol yield for all catalysts at pressures of 10, 30, and 50 bar and temperatures of 225, 250, 275, and 300 °C.

**Figure 8:**
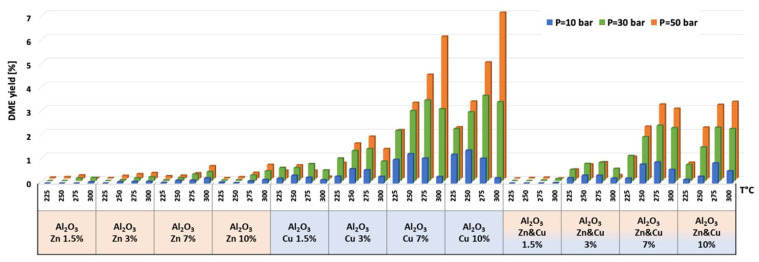
DME yield for all catalysts at pressures of 10, 30, and 50 bar and temperatures of 225, 250, 275, and 300 °C.

**Figure 9:**
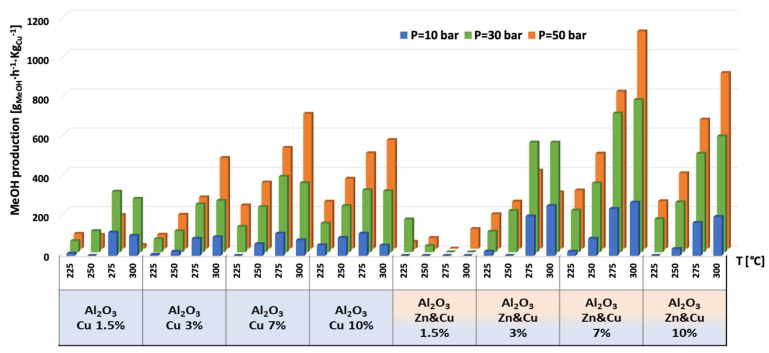
Production of methanol per gram of Cu for AlNFs-Cu and AlNFs-1Cu2.3Zn catalysts at pressures of 10, 30, and 50 bar and temperatures of 225, 250, 275, and 300 °C.

**Figure 10:**
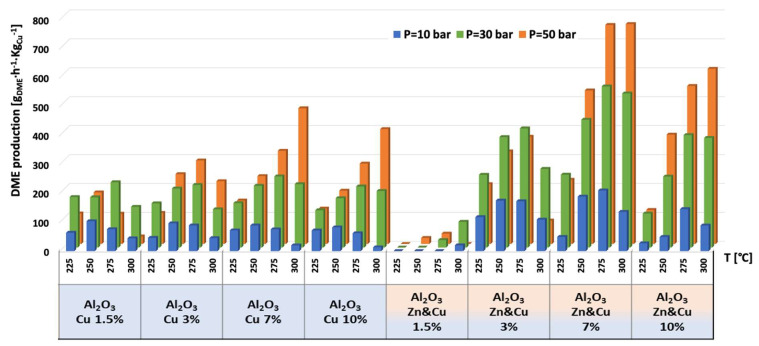
Production of DME per gram of Cu for AlNFs-Cu and AlNFs-1Cu2.3Zn catalysts at pressures of 10, 30, and 50 bar and temperatures of 225, 250, 275, and 300 °C.

**Figure 11:**
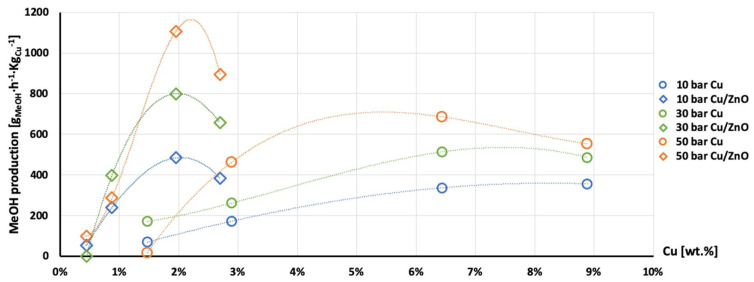
Production of methanol per gram of Cu as a function of Cu wt. % content in AlNFs-Cu (circle dots) and AlNFs-1Cu2.3Zn (rhombic dots) catalysts at pressures of 10 (blue), 30 (green), and 50 (orange) bar at temperature 300 °C.

**Figure 12:**
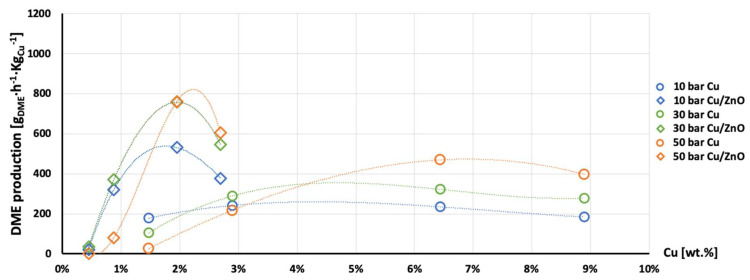
Production of DME per gram of Cu as a function of Cu wt. % content in AlNFs-Cu (circle dots) and AlNFs-1Cu2.3Zn (rhombic dots) catalysts at pressures of 10 (blue), 30 (green), and 50 (orange) bar at temperature 300 °C.

**Figure 13:**
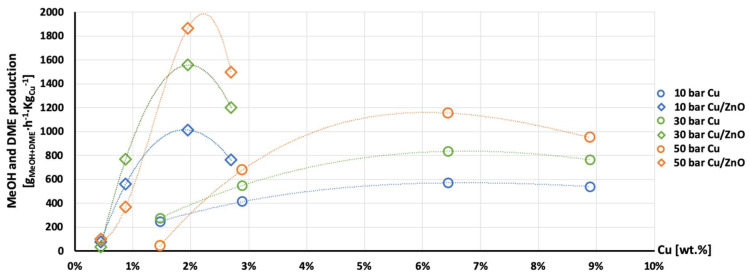
Production of the MeOH and DME per gram of Cu as a function of Cu wt. % content in AlNFs-Cu (circle dots) and AlNFs-1Cu2.3Zn (rhombic dots) catalysts at pressures of 10 (blue), 30 (green), and 50 (orange) bar at temperature 300 °C.

The catalytic results of CO_2_ hydrogenation to methanol and DME at 10, 30, and 50 bar and at 225, 250, 275, and 300 °C are presented in Figure 5, Figure 6, Figure 7, Figure 8, Figure 9, Figure 10, Figure 11, Figure 12 and Figure 13 and in Tables S5–S12. CO_2_ conversion as a function of temperature, pressure, and Cu, Zn, or Cu&Zn loadings in the catalysts is presented in Figure 5 and in Table S5. For all catalysts, CO_2_ conversion increased gradually with reaction temperature and pressure. The catalytic activity clearly depends on the catalysts’ metal loading. All AlNFs-Zn catalysts decorated with ZnO, showed relatively low catalytic activity, reflected by their low conversion and negligible methanol and DME yields, compared with Cu or mixed Cu/ZnO catalysts (Figure 5, Figure 7 and Figure 8 and Tables S5–S7). These results confirm that the reaction occurred on the surface of copper particles [62].The highest catalytic activity was obtained at 30 and 50 bar and 300 °C with the catalysts containing 7 and 10 wt% of Cu (catalysts AlNFs-Cu-7 and AlNFs-Cu-10). These two catalysts presented similar catalytic performance, reaching a CO_2_ conversion of 27–28% and total methanol and DME yield of 12.3-14%, which are close to the thermodynamic equilibrium values (29.6% and 13.7%, respectively) (Tables S5–S7 and Figures S9 and S10).

Selectivity to methanol and DME was strongly affected by temperature and pressure, as seen in Figure 6 for the AlNFs-Cu-10 catalyst. Increasing temperature correlated with a progressive decrease in oxygenated products’ (MeOH and DME) selectivity in favor of carbon monoxide. Increasing pressure correlated with a progressive increase in oxygenated products’ selectivity at the same temperature. However, as conversion increased dramatically with temperature (Figure 5), the overall contribution of reaction temperature was positive to obtain higher methanol and DME yields (Figure 7 and Figure 8), which as stated above, reached the thermodynamic limit (Figures S9 and S10). 

As stated above, the highest catalytic activity in formation of oxygenated products was observed in the tests of AlNFs-Cu catalysts with the highest Cu loadings (7 and 10 wt%), and the maximum yield of oxygenated products was obtained with AlNFs-Cu-10 catalyst (Figure 7 and Figure 8, Tables S6 and S7). At a pressure of 10 bar, a maximum selectivity to DME and methanol of ~40% was obtained at 225 °C. Increase in temperature at the same pressure (10 bar), led to a drastic decrease in DME and methanol selectivity, in favor of CO selectivity, that increased from 60% to 95% when the temperature increased from 225 °C to 300 °C. The maximum formation of oxygenated products was obtained at 50 bar. Under these conditions, the selectivity reached 60% at 225 °C and 43% at 300 °C. The total yield of methanol and DME products reached 6% at 225 °C and 14% at 300 °C.

The temperature at which maximum methanol and DME yields were obtained varied with the pressure and the catalyst type (Figure 7 and Figure 8). For AlNFs-Cu catalysts, maximum methanol yield at 10 and 30 bar were obtained at 275 °C, while at 50 bar, the optimal temperature shifted to 300 °C. In the case of DME production, the optimal formation temperature increased from 250 °C at 10 bar to 275 °C at 30 bar and to 300 °C at 50 bar. For AlNFs-ZnCu catalysts, 300 °C was the optimal temperature to obtain the maximum yield of methanol and 275 °C of DME, at all pressures.

According to thermodynamics, increasing the temperature favors the formation of methanol and, hence, raises the selectivity to methanol in the fraction of oxygenated products. At 30 bar, methanol selectivity increased from 17.6% to 40.2% when the temperature was raised from 225 °C to 300 °C. At 50 bar, the same tendency was observed (Figure S11). The catalytic results of AlNFs-Cu and AlNFs-1Cu2.3Zn catalysts presented in current study followed the above-described tendency with two exceptions. First, the chemical equilibrium was shifted towards methanol. Maximum selectivity to methanol in the fraction of oxygenated products obtained at 300 °C varied between 50 and 70%. Second, in the case of AlNFs-Cu-7 catalyst, temperature had no influence on methanol selectivity, and in the case of AlNFs-Cu-10 catalyst, increasing in temperature favored the formation of DME (Figure S11).

The highest production of methanol and DME per gram of Cu was obtained with the AlNFs-1Cu2.3Zn catalysts containing 7 and 10 wt% of Cu&Zn (Figure 9, Figure 10, Figure 11, Figure 12 and Figure 13 and Tables S10–S12). Up to 1106 g_methanol_Kg_Cu_^−1^∙h^−1^ and 760 g_DME_Kg_Cu_^−1^∙h^−1^ were obtained at 50 bar and 300 °C with AlNFs-1Cu2.3Zn-7 catalyst containing 7 wt% of Cu&Zn. The highest methanol and DME production per gram of Cu, for catalysts loaded with Cu only, obtained with the AlNFs-Cu-7 catalyst, reached 686 g_methanol_Kg_Cu_^−1^∙h^−1^ and 469 g_DME_Kg_Cu_^−1^∙h^−1^, at the same conditions. Increasing the loading to 10 wt% decreased production of oxygenated products per gram of Cu. Supported by TEM analysis (Figure 4), this effect can be due to sintering of Cu particles and decrease of surface active sites in the catalysts.

Correlation between Cu content and production of methanol and DME per gram of Cu presented in Figure 11, Figure 12 and Figure 13 show the synergy between Cu and ZnO in methanol and DME synthesis. According to Zander et al. [63], ZnO improves the dispersion, stabilizes the structure, and prevents the sintering of copper particles, which affects the adsorption properties of CO_2_ on the copper particles. According to Burch et al. [62], ZnO is capable of storing hydrogen in a readily available atomic form that diffuses to Cu and interacts with Cu formate molecule to form more stable hydrogenated intermediate of methanol synthesis. However, in a recent study, Wang et al. [64] used in situ FTIR to show that CO_2_ is adsorbed mainly on the Al_2_O_3_ and ZnO support, forming carbonate species, and that Cu facilitates H_2_ dissociation. Then, hydrogen atoms react with the adsorbed carbonates on the Al_2_O_3_ support and ZnO, transforming them to formate and additional intermediates. Additional and more powerful operando spectroscopy methods, such as ambient-pressure X-ray photoelectron spectroscopy (AP-XPS) and in situ (S)TEM, were recently used to characterize reactive interfacial structures in situ [65,66,67,68,69]. In future study, such operando spectroscopy characterization can be highly beneficial to study the catalytic mechanism in the current catalysts.

## 4. Conclusions

A series of electrospun alumina nanofibers decorated with different amounts of Cu, ZnO, or Cu/ZnO has been synthetized and studied as catalysts for CO_2_ hydrogenation to methanol and DME as a function of temperature and pressure using the high-throughput equipment of the REALCAT platform. Decoration was achieved by precipitation on nitrate-based solutions of the respective metals, which led to formation of metal nanosheets perpendicular to the fiber surface. We found that the catalytic activity depends on the metal loading and the Cu/Zn ratio. Nanofibers decorated with Cu and Cu/ZnO showed higher catalytic activity compared with nanofibers decorated with ZnO only. It was found that under the same reaction conditions, the hydrogenation rate of CO_2_ to methanol and DME is principally affected by the Cu ratio in catalyst and the synergy between Cu and ZnO. Maximal methanol production of ~1106 g_methanol_Kg_Cu_^−1^∙h^−1^ and DME production of 760 g_DME_Kg_Cu_^−1^∙h^−1^ were obtained for AlNFs-1Cu2.3Zn-7 catalyst containing 7 wt% of CuO and ZnO (at 300 °C, 50 bar). The combined methanol and DME yield was ~6%, and their combined production was 1866 g_MeOH+DME_Kg_Cu_^−1^∙h^−1^, which surpasses the best results published so far in the literature. It should be emphasized that in this work, the Cu/ZnO ratio and the metal loading had not been optimized. Such optimization has already begun and will be the subject of future work that is aimed to improve the production of methanol and DME. In addition, control over the degree of reduction of the initial CuO particles in the catalysts could also optimize our catalyst activity. For example, this could be achieved by tuning the reduction temperature or by varying the percentage of H_2_ in the reducing H_2_/N_2_ gas. Moreover, to achieve an understanding of the catalytic mechanism, operando spectroscopy characterization, methods such as AP-XPS and in situ (S)TEM, can be used.

## Supplementary Materials

The following supporting information can be downloaded at: https://www.mdpi.com/article/10.3390/nano13040635/s1, **Figure S1** Photos and scheme of Flowrence and reactors loading; **Figure S2** SEM images of raw alumina nanofibers in different magnifications, (a) 5 k and (b) 15 k; **Figure S3** SEM images of calcined (at 973 K) alumina nanofibers in different magnifications, (a) 5 k and (b) 50 k. (c) is a histogram of fibers’ diameter (94 fibers); **Figure S4:** Histograms of alumina nanofibers’ diameter. (a) raw nanofibers (87 fibers) (b) calcined nanofibers (94 fibers); **Figure S5.** HRSEM images of AlNFs decorated by 10 (a, b), 7 (c, d) 3 (e, f) and 1.5 (g, h) wt% of Zn&Cu in the form of ZnO&CuO, in a wt. ratio of 2.3 Zn/Cu; **Figure S6:** N_2_ physisorption isotherms of calcined alumina nanofibers AlNFs (a) and few calcined alumina nanofiber decorated with Cu, Zn and Cu\Zn: AlNFs-Cu catalysts decorated with 1.5 wt% (b), 7 wt% (c) and 10 wt% (d) Cu, AlNFs-1Cu2.3Zn catalysts decorated with 1.5 wt% (e) and 7 wt% (f) Cu and Zn (Cu/Zn=1:2.3), AlNFs-Zn catalyst decorated with 7 wt% Zn (g); **Figure S7:** SEM images of pre-calcined AlNFs decorated by 7 wt% Zn and Cu (in a wt. ratio of 2.3 Zn/Cu) in the form of Zn(NO_3_)_2_ and Cu(NO_3_)_2_. (a)–(d) are magnifications of 5 k, 15 k, 50 k and 150 k, respectively; **Figure S8:** SEM-EDS results of decorated alumina nanofibers: (a) AlNFs-Zn-7–alumina nanofiber decorated with 7 wt% of Zn (in form of ZnO); (b) AlNFs-Cu-7–alumina nanofiber decorated with 7 wt% of Cu (in form of CuO); (b) AlNFs-1Cu2.3Zn-7–alumina nanofiber decorated with 7 wt% of Cu and Zn (in form of CuO and ZnO) in a wt. ratio 1:2.3; **Figure S9:** Effect of temperature on CO_2_ conversion and yields to Methanol, Dimethyl ether and CO at equilibrium at 30 bar pressure and H_2_/CO_2_=4 ratio; **Figure S10:** Effect of temperature on CO_2_ conversion and yields to Methanol, Dimethyl ether and CO at equilibrium at 50 bar pressure and H_2_/CO_2_=4 ratio; **Figure S11.** Selectivity to methanol in oxygenated products (MeOH and DME) fraction for some AlNFs-Cu and AlNFs-1Cu2.3Zn catalysts at pressures of 30 and 50 bar and temperatures of 225, 250, 275, 300 ºC; **Table S1** Comparison of the results of the present study to those of the literature; **Table S2** Cu, Zn or Cu&Zn weight loadings on Electorspun Alumina nanofibers in each catalyst and BET SSA for chosen catalysts; **Table S3** ICP and XRF results in wt%. Wt. % is defined as Me/(Al_2_O_3_+MeO), when Me can be Zn, Cu or both. Deviation superior to 1% from expected values are marked in orange; **Table S4:** particles’ sizes of AlNFs-1Cu2.3Zn-10 alumina nanofibers decorated with 10 wt% of Cu and Zn in a wt. ratio 1:2.3 as calculated by Scherrer equation; **Table S5** CO_2_ conversions for all catalysts at pressures of 10, 30, 50 bar and temperatures of 225, 250, 275, 300 °C; **Table S6** Methanol yield for all catalysts at pressures of 10, 30, 50 bar and temperatures of 225, 250, 275, 300 °C; **Table S7** DME yield for all catalysts at pressures of 10, 30, 50 bar and temperatures of 225, 250, 275, 300 °C; **Table S8** CO yield for all catalysts at pressures of 10, 30, 50 bar and temperatures of 225, 250, 275, 300 °C; **Table S9** CH_4_ yield for all catalysts at pressures of 10, 30, 50 bar and temperatures of 225, 250, 275, 300 °C; **Table S10** Space time yield of methanol per gram of Cu for AlNFs-Cu and AlNFs-1Cu2.3Zn catalysts at pressures of 10, 30, 50 bar and temperatures of 225, 250, 275, 300 °C; **Table S11** Space time yield to DME per gram of Cu for AlNFs-Cu and AlNFs-1Cu2.3Zn catalysts at pressures of 10, 30, 50 bar and temperatures of 225, 250, 275, 300 °C; **Table S12** Space time yield to Methanol and DME per gram of Cu for AlNFs-Cu and AlNFs-1Cu2.3Zn catalysts at pressures of 10,30,50 bar and temperatures of 225, 250, 275, 300 °C. References [70,71] are cited in the Supplementary Materials.

## References

[1] Jiang X., Nie X., Guo X., Song C., Chen J.G. (2020). Recent Advances in Carbon Dioxide Hydrogenation to Methanol via Heterogeneous Catalysis. Chem. Rev..

[2] Appel A.M., Bercaw J.E., Bocarsly A.B., Dobbek H., Dubois D.L., Dupuis M., Ferry J.G., Fujita E., Hille R., Kenis P.J.A. (2013). Frontiers, Opportunities, and Challenges in Biochemical and Chemical Catalysis of CO_2_ Fixation. Chem. Rev..

[3] Lindsey R., Dlugokencky E. Climate Change: Atmospheric Carbon Dioxide|NOAA Climate.Gov. https://www.climate.gov/news-features/understanding-climate/climate-change-atmospheric-carbon-dioxide.

[4] Saeidi S., Aishah N., Amin S., Reza M. (2014). Hydrogenation of CO_2_ to Value-Added Products—A Review and Potential Future Developments. Biochem. Pharmacol..

[5] Hu B., Frueh S., Garces H.F., Zhang L., Aindow M., Brooks C., Kreidler E., Suib S.L. (2013). Selective Hydrogenation of CO_2_ and CO to Useful Light Olefins over Octahedral Molecular Sieve Manganese Oxide Supported Iron Catalysts. Appl. Catal. B Environ..

[6] Goeppert A., Czaun M., Jones J.P., Surya Prakash G.K., Olah G.A. (2014). Recycling of Carbon Dioxide to Methanol and Derived Products-Closing the Loop. Chem. Soc. Rev..

[7] Olah G.A., Prakash G.K.S., Goeppert A. (2011). Anthropogenic Chemical Carbon Cycle for a Sustainable Future. J. Am. Chem. Soc..

[8] Novikov A.S., Kuznetsov M.L., Rocha B.G.M., Pombeiro A.J.L., Shul’pin G.B. (2016). Oxidation of Olefins with H_2_O_2_ Catalysed by Salts of Group III Metals (Ga, In, Sc, y and La): Epoxidation versus Hydroperoxidation. Catal. Sci. Technol..

[9] Nieminen H., Laari A., Koiranen T. (2019). CO_2_ Hydrogenation to Methanol by a Liquid-Phase Process with Alcoholic Solvents: A Techno-Economic Analysis. Processes.

[10] Dang S., Yang H., Gao P., Wang H., Li X., Wei W., Sun Y. (2019). A Review of Research Progress on Heterogeneous Catalysts for Methanol Synthesis from Carbon Dioxide Hydrogenation. Catal. Today.

[11] Ott J., Gronemann V., Pontzen F., Fiedler E., Grossmann G., Kersebohm D.B., Weiss G.N., Witte C., Wiley-VCH (2012). Methanol. Ullmann’s Encyclopedia of Industrial Chemistry.

[12] Bowker M. (2019). Methanol Synthesis from CO_2_ Hydrogenation. ChemCatChem.

[13] Yang Y., Mei D., Peden C.H.F., Campbell C.T., Mims C.A. (2015). Surface-Bound Intermediates in Low-Temperature Methanol Synthesis on Copper: Participants and Spectators. ACS Catal..

[14] Ma J., Sun N., Zhang X., Zhao N., Xiao F., Wei W., Sun Y. (2009). A Short Review of Catalysis for CO_2_ Conversion. Catal. Today J..

[15] Joshi J.B. (2014). Catalytic Carbon Dioxide Hydrogenation to Methanol: A Review of Recent Studies. Chem. Eng. Res. Des..

[16] Grabow L.C., Mavrikakis M. (2011). Mechanism of Methanol Synthesis on Cu through CO_2_ and CO Hydrogenation. ACS Catal..

[17] Qi T., Zhao Y., Chen S., Li W., Guo X., Zhang Y., Song C. (2021). Bimetallic Metal Organic Framework-Templated Synthesis of a Cu-ZnO/Al2O3 Catalyst with Superior Methanol Selectivity for CO_2_ Hydrogenation. Mol. Catal..

[18] Kattel S., Chen J.G., Liu P. (2017). Active Sites for CO_2_ Hydrogenation to Methanol on Cu/ZnO Catalysts. Science.

[19] Behrens M., Studt F., Kasatkin I., Kühl S., Hävecker M., Abild-pedersen F., Zander S., Girgsdies F., Kurr P., Kniep B. (2012). The Active Site of Methanol Synthesis over Cu/ZnO/Al_2_O_3_ Industrial Catalysts. Science.

[20] Spencer M.S. (1999). The Role of Zinc Oxide in Cu/ZnO Catalysts for Methanol Synthesis and the Water-Gas Shift Reaction. Top. Catal..

[21] Martin O., Mondelli C., Curulla-ferre D., Drouilly C., Hauert R., Pe J. (2015). Zinc-Rich Copper Catalysts Promoted by Gold for Methanol Synthesis. ACS Catal..

[22] Li M.M., Zeng Z., Liao F., Hong X., Chi S., Tsang E. (2016). Enhanced CO_2_ Hydrogenation to Methanol over CuZn Nanoalloy in Ga Modified Cu / ZnO Catalysts. J. Catal..

[23] Ren S., Fan X., Shang Z., Shoemaker W.R., Ma L., Wu T., Li S., Klinghoffer N.B., Yu M., Liang X. (2020). Enhanced Catalytic Performance of Zr Modified CuO/ZnO/Al_2_O_3_ Catalyst for Methanol and DME Synthesis via CO_2_ Hydrogenation. J. CO_2_ Util..

[24] Li S., Guo L., Ishihara T. (2020). Hydrogenation of CO_2_ to Methanol over Cu/AlCeO Catalyst. Catal. Today.

[25] Grunwaldt J.D., Molenbroek A.M., Topsøe N.Y., Topsøe H., Clausen B.S. (2000). In Situ Investigations of Structural Changes in Cu/ZnO Catalysts. J. Catal..

[26] An X., Zuo Y., Zhang Q., Wang J. (2009). Methanol Synthesis from CO_2_ Hydrogenation with a Cu/Zn/Al/Zr Fibrous Catalyst. Chin. J. Chem. Eng..

[27] Tisseraud C., Comminges C., Belin T., Ahouari H., Soualah A. (2015). The Cu–ZnO Synergy in Methanol Synthesis from CO_2_, Part 2: Origin of the Methanol and CO Selectivities Explained by Experimental Studies and a Sphere Contact Quantification Model in Randomly Packed Binary Mixtures on Cu–ZnO Coprecipitate Catalysts. J. Catal..

[28] Le Valant A., Comminges C., Tisseraud C., Canaff C., Pinard L., Pouilloux Y. (2015). The Cu-ZnO Synergy in Methanol Synthesis from CO_2_, Part 1: Origin of Active Site Explained by Experimental Studies and a Sphere Contact Quantification Model on Cu + ZnO Mechanical Mixtures. J. Catal..

[29] Tisseraud C., Comminges C., Pronier S., Pouilloux Y., Valant A. (2016). Le The Cu-ZnO Synergy in Methanol Synthesis Part 3: Impact of the Composition of a Selective Cu @ ZnO x Core—Shell Catalyst on Methanol Rate Explained by Experimental Studies and a Concentric Spheres Model. J. Catal..

[30] Nakamura J., Nakamura I., Uchijima T., Kanai Y., Watanabe T., Saito M., Fujitani T. (1996). A Surface Science Investigation of Methanol Synthesis over a Zn-Deposited Polycrystalline Cu Surface. J. Catal..

[31] Lam E., Corral-Pérez J.J., Larmier K., Noh G., Wolf P., Comas-Vives A., Urakawa A., Copéret C. (2019). CO_2_ Hydrogenation on Cu/Al_2_O_3_: Role of the Metal/Support Interface in Driving Activity and Selectivity of a Bifunctional Catalyst. Angew. Chemie Int. Ed..

[32] Pontzen F., Liebner W., Gronemann V., Rothaemel M., Ahlers B. (2011). CO_2_-Based Methanol and DME-Efficient Technologies for Industrial Scale Production. Catal. Today.

[33] Sahebdelfar S., Bijani P.M., Yaripour F. (2022). Deactivation Kinetics of γ-Al_2_O_3_ Catalyst in Methanol Dehydration to Dimethyl Ether. Fuel.

[34] Azizi Z., Rezaeimanesh M., Tohidian T., Rahimpour M.R. (2014). Dimethyl Ether: A Review of Technologies and Production Challenges. Chem. Eng. Process. Process Intensif..

[35] Carvalho D.F., Almeida G.C., Monteiro R.S., Mota C.J.A. (2020). Hydrogenation of CO_2_ to Methanol and Dimethyl Ether over a Bifunctional Cu·ZnO Catalyst Impregnated on Modified γ-Alumina. Energy Fuels.

[36] Mota N., Ordoñez E.M., Pawelec B., Fierro J.L.G., Navarro R.M. (2021). Direct Synthesis of Dimethyl Ether from CO_2_: Recent Advances in Bifunctional/Hybrid Catalytic Systems. Catalysts.

[37] Yao L., Shen X., Pan Y., Peng Z. (2019). Synergy between Active Sites of Cu-In-Zr-O Catalyst in CO_2_ Hydrogenation to Methanol. J. Catal..

[38] Samson K., Śliwa M., Socha R.P., Góra-Marek K., Mucha D., Rutkowska-Zbik D., Paul J.-F., Ruggiero-Mikołajczyk M., Grabowski R., Słoczyński J. (2014). Influence of ZrO_2_ Structure and Copper Electronic State on Activity of Cu/ZrO_2_ Catalysts in Methanol Synthesis from CO_2_ † ’. ACS Catal..

[39] Men Y.L., Liu Y., Wang Q., Luo Z.H., Shao S., Li Y.B., Pan Y.X. (2019). Highly Dispersed Pt-Based Catalysts for Selective CO_2_ Hydrogenation to Methanol at Atmospheric Pressure. Chem. Eng. Sci..

[40] Kolmakov A., Moskovits M. (2004). Chemical Sensing and Catalysis by One-Dimensional Metal-Oxide Nanostructures. Annu. Rev. Mater. Res..

[41] Khajavi R., Abbasipour M. (2012). Electrospinning as a Versatile Method for Fabricating Coreshell, Hollow and Porous Nanofibers. Sci. Iran..

[42] Xue J., Wu T., Dai Y., Xia Y. (2019). Electrospinning and Electrospun Nanofibers: Methods, Materials, and Applications. Chem. Rev..

[43] Elishav O., Poliak L., Naamat I., Beilin V., Shter G.E., Grader G.S. (2019). Lamellar-like Electrospun Mesoporous Ti-Al-O Nanofibers. Materials.

[44] Elishav O., Beilin V., Shter G.E., Dinner O., Halperin V., Grader G.S. (2017). Formation of Core-Shell Mesoporous Ceramic Fibers. J. Am. Ceram. Soc..

[45] Yang A., Tao X., Pang G.K.H., Siu K.G.G. (2008). Preparation of Porous Tin Oxide Nanobelts Using the Electrospinning Technique. J. Am. Ceram. Soc..

[46] Halperin V., Shter G.E., Gelman V., Peselev D.M., Mann-Lahav M., Grader G.S. (2015). Catalytic Activity of Electrospun Ag and Ag/Carbon Composite Fibres in Partial Methanol Oxidation. Catal. Sci. Technol..

[47] Bauer A., Lee K., Song C., Xie Y., Zhang J., Hui R. (2010). Pt Nanoparticles Deposited on TiO_2_ Based Nanofibers: Electrochemical Stability and Oxygen Reduction Activity. J. Power Sources.

[48] Jiménez-Morales I., Cavaliere S., Jones D., Rozière J. (2018). Strong Metal-Support Interaction Improves Activity and Stability of Pt Electrocatalysts on Doped Metal Oxides. Phys. Chem. Chem. Phys..

[49] Sun Y., Chen L., Bao Y., Wang G., Zhang Y., Fu M., Wu J., Ye D. (2017). Roles of Nitrogen Species on Nitrogen-Doped CNTs Supported Cu-ZrO_2_ System for Carbon Dioxide Hydrogenation to Methanol. Catal. Today.

[50] Liu Q., Zhu J., Zhang L., Qiu Y. (2018). Recent Advances in Energy Materials by Electrospinning. Renew. Sustain. Energy Rev..

[51] Condon J.B. (2020). An Overview and Some Uninteresting History of Physisorption. Surface Area and Porosity Determinations by Physisorption.

[52] Tian M., Yao L., Han A., Zhu X., Chen C., Ye M., Chen X. (2020). Near-Infrared Re Fl Ectance and Thermal Insulating Performance of Mo-Doped Bi_2_WO_6_ with 3D Hierarchical Fl Ower-like Structure as Novel Ceramics Pigment. Ceram. Int..

[53] Lu Z., Zhao Z., Yang L., Wang S., Liu H., Feng Y., Zhao Y., Feng F. (2019). A Simple Method for Synthesis of Highly Efficient Flower-like—SnO_2_ Photocatalyst Nanocomposites. J. Mater. Sci. Mater. Electron..

[54] Li J., Liu H. (2020). Facile Fabrication of Fe-Doped Si-C-N Ceramic Microspheres with Fl Ower-like Morphology and the Infrared Extinction Property. J. Sol-Gel Sci. Technol..

[55] Landman A., Hadash S., Shter G.E., Ben-Azaria A., Dotan H., Rothschild A., Grader G.S. (2021). High Performance Core/Shell Ni/Ni(OH)2 Electrospun Nanofiber Anodes for Decoupled Water Splitting. Adv. Funct. Mater..

[56] Leonova Y.O., Sevostyanov M.A., Mezentsev D.O., Khayrutdinova D.R., Lysenkov A.S. (2021). Effect of the Synthesis Temperature on the Phase Composition of Al_2_O_3_. J. Phys. Conf. Ser..

[57] Małecka B., Łącz A., Drozdz E., Małecki A. (2015). Thermal Decomposition of D-Metal Nitrates Supported on Alumina. J. Therm. Anal. Calorim..

[58] Ramesh S., Aw K.L., Ting C.H., Tan C.Y., Sopyan I., Teng W.D. (2008). Effect of Copper Oxide on the Sintering of Alumina Ceramics. Adv. Mater. Res..

[59] El-Mehalawy N., Awaad M., Eliyan T., Abd-Allah M.A., Naga S.M. (2018). Electrical Properties of ZnO/Alumina Nano Composites for High Voltage Transmission Line Insulator. J. Mater. Sci. Mater. Electron..

[60] Milak P., Minatto F.D., Faller C., De Noni A., Klegues Montedo O.R. (2015). The Influence of Dopants in the Grain Size of Alumina—A Review. Mater. Sci. Forum.

[61] Biotteau-Deheuvels K., Zych L., Gremillard L., Chevalier J. (2012). Effects of Ca-, Mg- and Si-Doping on Microstructures of Alumina-Zirconia Composites. J. Eur. Ceram. Soc..

[62] Burch R., Golunski S.E., Spencer M.S. (1990). The Role of Hydrogen in Methanol Synthesis over Copper Catalysts. Catal. Lett..

[63] Zander S., Kunkes E.L., Schuster M.E., Schumann J., Weinberg G., Teschner D., Jacobsen N., Schlögl R., Behrens M. (2013). The Role of the Oxide Component in the Development of Copper Composite Catalysts for Methanol Synthesis. Angew. Chemie Int. Ed..

[64] Wang L., Etim U.J., Zhang C., Amirav L., Zhong Z. (2022). CO_2_ Activation and Hydrogenation on Cu-ZnO/Al_2_O_3_ Nanorod Catalysts: An In Situ FTIR Study. Nanomaterials.

[65] Ren Y., Yuan K., Zhou X., Sun H., Wu K., Bernasek S.L., Chen W., Xu G.Q. (2018). Catalytic Intermediates of CO_2_ Hydrogenation on Cu(111) Probed by In Operando Near-Ambient Pressure Technique. Chem. A Eur. J..

[66] Wu C.H., Liu C., Su D., Xin H.L., Fang H.T., Eren B., Zhang S., Murray C.B., Salmeron M.B. (2019). Bimetallic Synergy in Cobalt–Palladium Nanocatalysts for CO Oxidation. Nat. Catal..

[67] Kim T.S., Kim J., Song H.C., Kim D., Jeong B., Lee J., Shin J.W., Ryoo R., Park J.Y. (2020). Catalytic Synergy on PtNi Bimetal Catalysts Driven by Interfacial Intermediate Structures. ACS Catal..

[68] Lee H., Lim J., Lee C., Back S., An K., Shin J.W., Ryoo R., Jung Y., Park J.Y. (2018). Boosting Hot Electron Flux and Catalytic Activity at Metal-Oxide Interfaces of PtCo Bimetallic Nanoparticles. Nat. Commun..

[69] Xin H.L., Alayoglu S., Tao R., Genc A., Wang C.M., Kovarik L., Stach E.A., Wang L.W., Salmeron M., Somorjai G.A. (2014). Revealing the Atomic Restructuring of Pt-Co Nanoparticles. Nano Lett..

[70] Navarro-Jaén S., Virginie M., Thuriot-Roukos J., Wojcieszak R., Khodakov A.Y. (2022). Structure–Performance Correlations in the Hybrid Oxide-Supported Copper–Zinc SAPO-34 Catalysts for Direct Synthesis of Dimethyl Ether from CO_2_. J. Mater. Sci..

[71] Navarro-Jaén S., Virginie M., Morin J.C., Thuriot-Roukos J., Wojcieszak R., Khodakov A.Y. (2022). Hybrid Monometallic and Bimetallic Copper-Palladium Zeolite Catalysts for Direct Synthesis of Dimethyl Ether from CO_2_. New J. Chem..

