# Supplementary material for: Performance of Cu/ZnO Nanosheets on Electrospun Al2O3 Nanofibers in CO2 Catalytic Hydrogenation to Methanol and Dimethyl Ether
Source: Nanomaterials (Basel). 2023 Feb 5;13(4):635. doi: 10.3390/nano13040635 (PMC9967565; doi:10.3390/nano13040635)
Supplement: Supplementary file 1 [file nanomaterials-13-00635-s001.zip › nanomaterials-2202003-supplementary.pdf]

Table S1 Comparison of the results of the present study to those of the literature.

Reference	CuO:ZnO ratio in the catalyst	Support type	Synthesis method	BET SSA [m^2/g]	GHSV or WHSV	Tests conditions	MeOH productivity $g_{MeOH}/kg_{Cu}/h$	DME productivity $g_{DME}/kg_{Cu}/h$	Total productivity $g_{MeOH+DME}/kg_{Cu}/h$
**Wang *et al.* 2022 [1]**	3 wt% of Cu/ZnO with 2.46 wt.% of Cu	n-Al_2O_3 nanorods	incipient wetness impregnation method	89.4	GHSV=7 800 mL/g_{cat}/h	CO_2:H_2 (mol.) 1:3 30 bar 300°C	1 706	0	1 706
**Navarro-Jaen *et al.* 2022 [2]**	Cu/ZnO/Al_2O_3 (wt%)= 38.6/22/6.3	SAPO-34 (SiO_2/Al_2O_3=0.5)	co-precipitation with following mechanical mixture with support	-	GHSV=3 000 mL/g_{cat}/h	CO_2:H_2 (mol.) 1:3 10 bar 220°C	63	99	162
**Lam *et al.* 2019 [3]**	3.8 wt% of Cu	γ-alumina	SOMC, grafting of Cu on γ-alumina	141	GHSV=24 000 mL/g_{cat}/h	CO_2:H_2:N_2 (mol.) 1:3:1 25 bar 230 °C	440	350	890
**Navarro-Jaen *et al.* 2022 [4]**	7 wt% of Cu	γ-alumina/ZSM-5	co-precipitation	208.6	GHSV=3 000 mL/g_{cat}/h	CO_2:H_2:N_2 (mol.) 1:3:1 20 bar 260°C	76	98	174
**Carvalho *et al.* 2020 [5]**	8 wt% of Cu/ZnO (Cu/Zn=1:1) with 5.4 wt% of Cu	γ-alumina/SiO_2	wet impregnation	219	WHSV=0.23 1/h	CO_2:H_2 (mol.) 1:3 50 bar 290°C	176	185	362
**Present study**	7 wt% Cu/Zu (Cu/Zn=1:2.3) with 2.69 wt% of Cu	Electrospun alumina nanofibers AlNFs	wet impregnation	~300	GHSV=2 626 mL/g_{cat}/h WHSV=1 g_{CO2}/g_{cat}/h	CO_2:H_2:He (mol.) 1:4:0.16 50 bar 300°C	1106	760	1866

Table S2 Cu, Zn or Cu&Zn weight loadings on Electorspun Alumina nanofibers in each catalyst and BET SSA for chosen catalystS

Catalyst N°	Catalysts type	Catalyst Ref.	Metal loading of electrospun Al_2O_3 nanofibers [wt%]	BET SSA [m^2/g]
1	AlNFs-Zn catalysts	AlNFs-Zn-1.5	1.5% Zn	
2		AlNFs-Zn-3	3% Zn	
3		AlNFs-Zn-7	7% Zn	258
4		AlNFs-Zn-10	10% Zn	
5	AlNFs-Cu catalysts	AlNFs-Cu-1.5	1.5% Cu	326
6		AlNFs-Cu-3	3% Cu	
7		AlNFs-Cu-7	7% Cu	278
8		AlNFs-Cu-10	10% Cu	201
9	AlNFs-CuZn catalysts	AlNFs-1Cu2.3Zn-1.5	1.5% Cu:Zn=1:2.3	303
10		AlNFs-1Cu2.3Zn-3	3% Cu:Zn=1:2.3	
11		AlNFs-1Cu2.3Zn-7	7% Cu:Zn=1:2.3	260
12		AlNFs-1Cu2.3Zn-10	10% Cu:Zn=1:2.3	

Details of conversions', selectivity's, yields' and thermodynamic calculations

CO_2 conversion, X_{CO_2}, was determined using the following equation:

$$X_{CO_2} = \frac{F_{CO_2 inlet} - F_{CO_2 outlet}}{F_{CO_2 inlet}} \times 100\%$$

where $F_{CO_2 inlet}$ and $F_{CO_2 outlet}$ are the molar flowrates of CO_2 at the inlet and outlet of the reactor (*mol/min*), respectively.

Selectivity was calculated using the following equation:

$$S_i = \frac{F_{i\ outlet} \times N_i}{\sum_i^N F_{i\ outlet} \times N_i} \times 100\%$$

Where i is a product of reaction (namely CO, CH_4, methanol or DME), $F_{i\ outlet}$ the molar flowrate of i at the outlet of the reactor (*mol/min*) and N_i the number of carbon atoms in the product i.

Yields were calculated using the following equation:

$$Y_i = \frac{F_{i\ outlet} \times N_i}{F_{CO_2 inlet} - F_{CO_2 outlet}} \times 100\%$$

Methanol and DME Space Time Yield (P_{MeOH} and P_{DME}) was calculated per mass of metal (Zn, Cu or Zn+Cu) present in the catalyst ($g_{MeOH}\ kg_{Me}^{-1}h^{-1}$) using the following equation:

$$P_i = \frac{F_{i\ outlet} \times M_i}{m_{Me}}$$

Where i is MeOH or DME, M is the molar mass and m_{Me} the total mass of metals in the catalyst.

Thermodynamic calculations to predict the limit of CO_2 conversion and products yields in a given set of operating conditions were made using the ASPEN Plus software with a Predictive Soave-Redlich-Kwong equation of state. A Gibbs reactor was used and calculations were based on the minimization of the Gibbs energy of the defined thermodynamic system containing CO_2, H_2, Methanol (CH_3OH), Dimethyl Ether (DME) C_2H_6O, H_2O and CO.

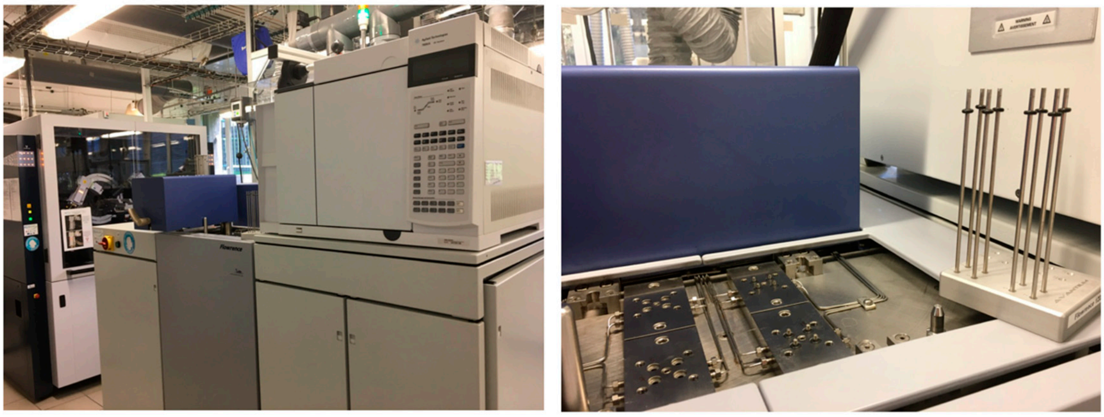

Photos of the Flowrence unit with its 4 blocks of 4 reactors

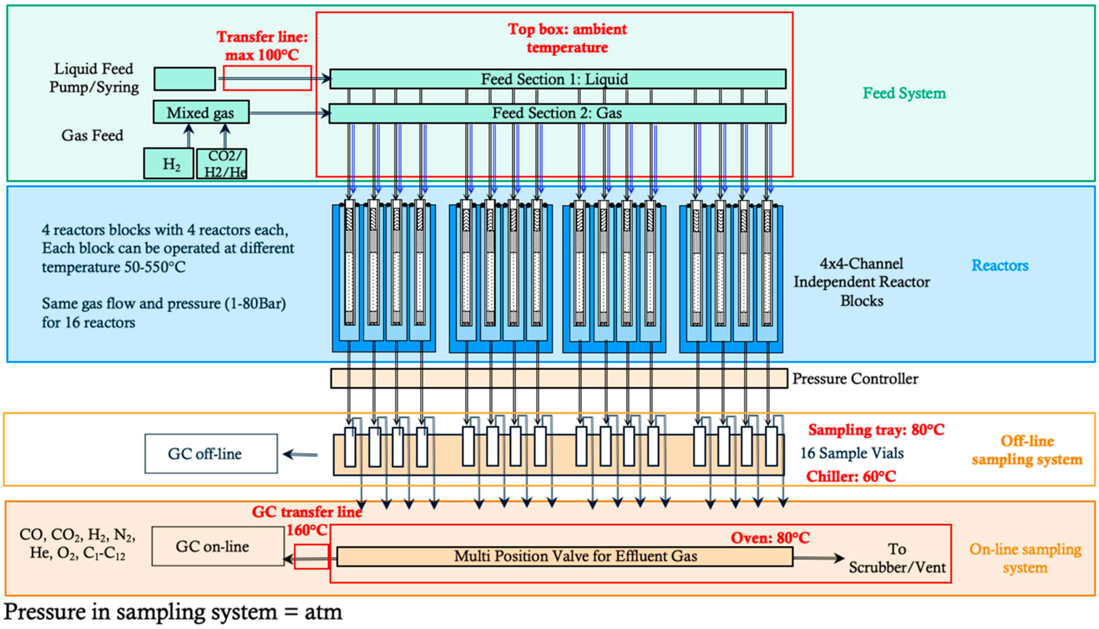

Scheme of the Flowrence unit

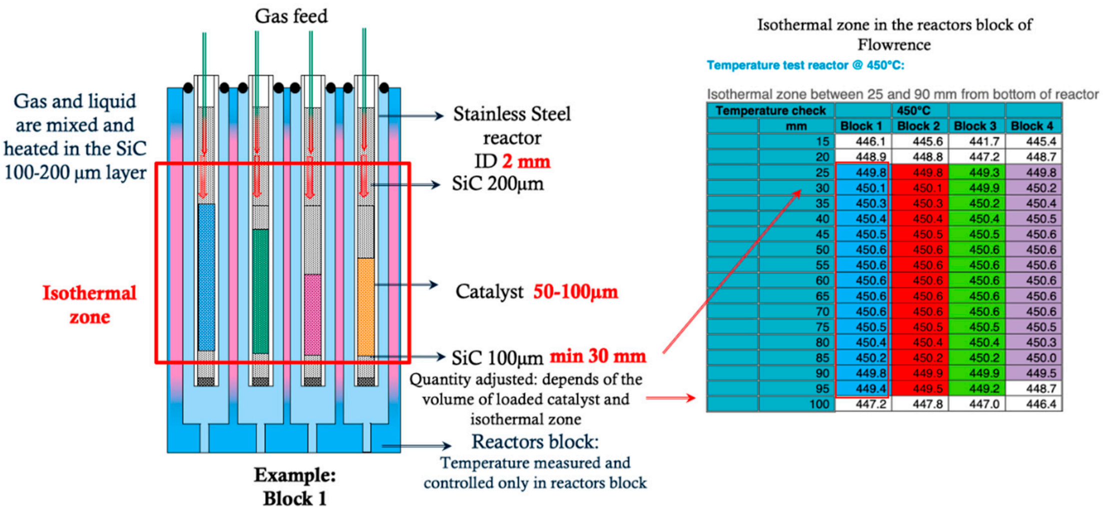

Isothermal zone in the reactors block of Flowrence

Temperature test reactor @ 450°C:

Isothermal zone between 25 and 90 mm from bottom of reactor

Temperature check			450°C		
	mm	Block 1	Block 2	Block 3	Block 4
	15	446.1	445.6	441.7	445.4
	20	448.9	448.8	447.2	448.7
	25	449.8	449.8	449.3	449.8
	30	450.1	450.1	449.9	450.2
	35	450.3	450.3	450.2	450.4
	40	450.4	450.4	450.4	450.5
	45	450.5	450.5	450.5	450.6
	50	450.6	450.6	450.6	450.6
	55	450.6	450.6	450.6	450.6
	60	450.6	450.6	450.6	450.6
	65	450.6	450.6	450.6	450.6
	70	450.6	450.6	450.6	450.6
	75	450.5	450.5	450.5	450.5
	80	450.4	450.4	450.4	450.3
	85	450.2	450.2	450.2	450.0
	90	449.8	449.9	449.9	449.5
	95	449.4	449.5	449.2	448.7
	100	447.2	447.8	447.0	446.4

Reactors loading

Figure S1 Photos and scheme of Flowrence and reactors loading.

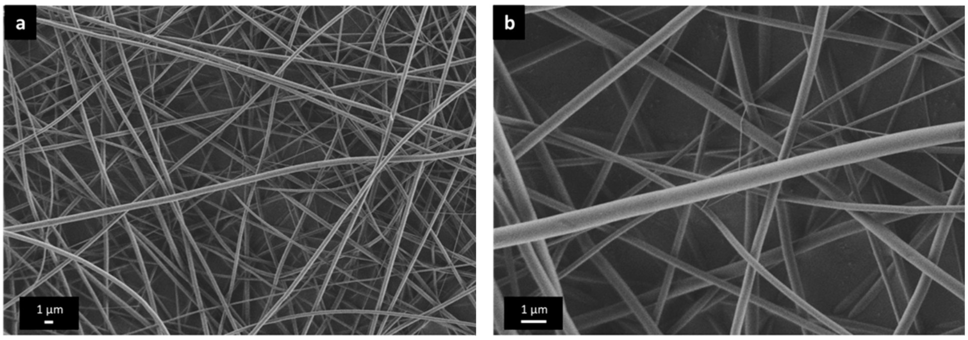

Figure S2 SEM images of raw alumina nanofibers in different magnifications, (a) 5 k and (b) 15 k.

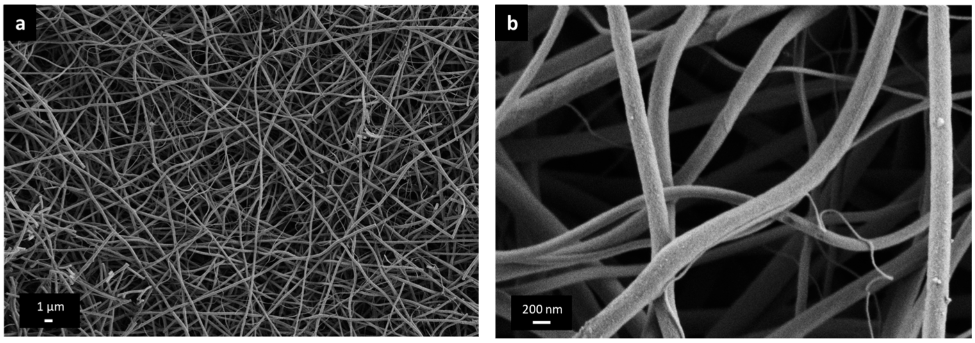

Figure S3 SEM images of calcined (at 973 K) alumina nanofibers in different magnifications, (a) 5 k and (b) 50 k. (c) is a histogram of fibers' diameter (94 fibers).

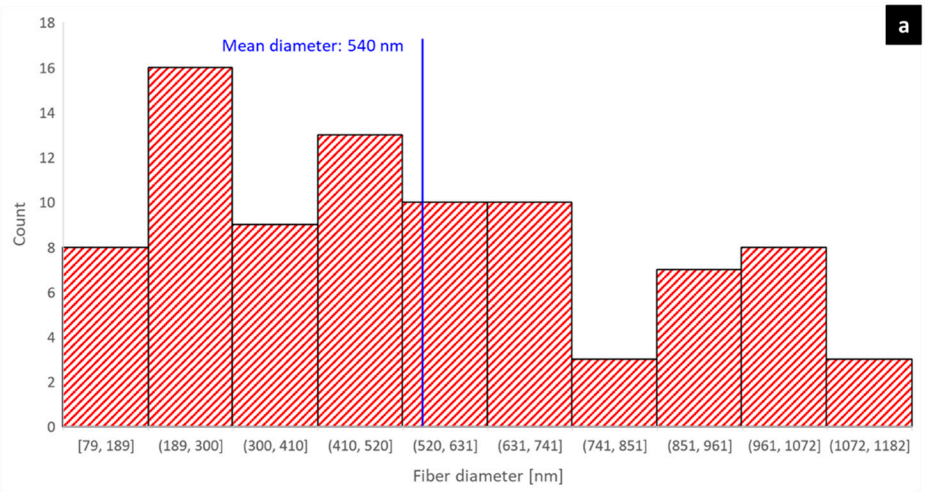

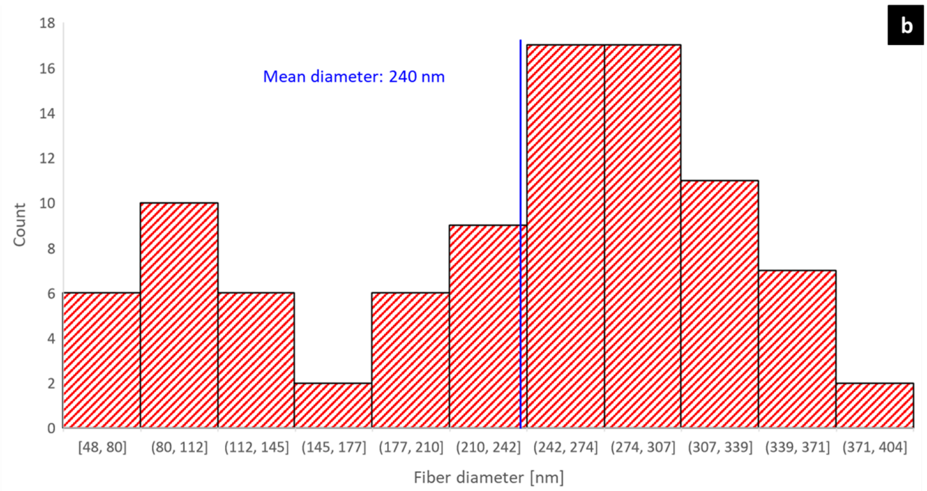

Figure S4: Histograms of alumina nanofibers' diameter. (a) raw nanofibers (87 fibers) (b) calcined nanofibers (94 fibers).

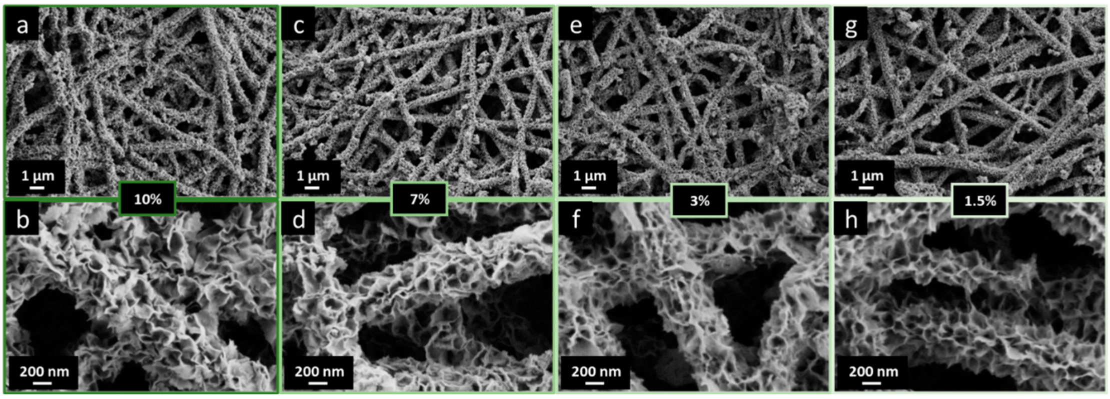

Figure S5. HRSEM images of AlNFs decorated by 10 (a, b), 7 (c, d) 3 (e, f) and 1.5 (g,h) wt% of Zn&Cu in the form of ZnO&CuO, in a wt. ratio of 2.3 Zn/Cu.

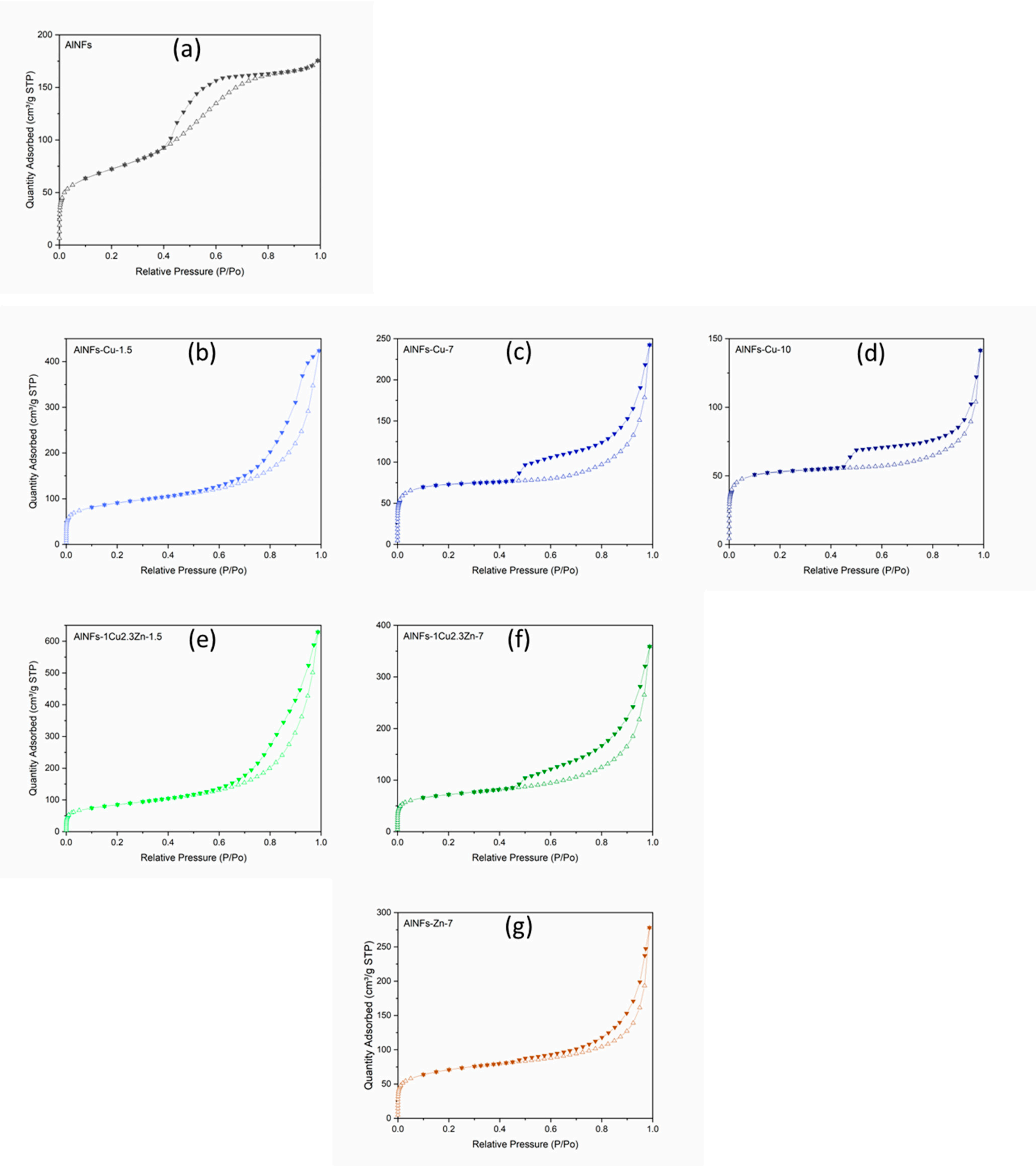

Figure S6: N_2 physisorption isotherms of calcined alumina nanofibers AlNFs (a) and few calcined alumina nanofiber decorated with Cu, Zn and Cu\Zn: AlNFs-Cu catalysts decorated with 1.5 wt% (b), 7 wt% (c) and 10 wt% (d) Cu, AlNFs-1Cu2.3Zn catalysts decorated with 1.5 wt% (e) and 7 wt% (f) Cu and Zn (Cu/Zn=1:2.3), AlNFs-Zn catalyst decorated with 7 wt% Zn (g).

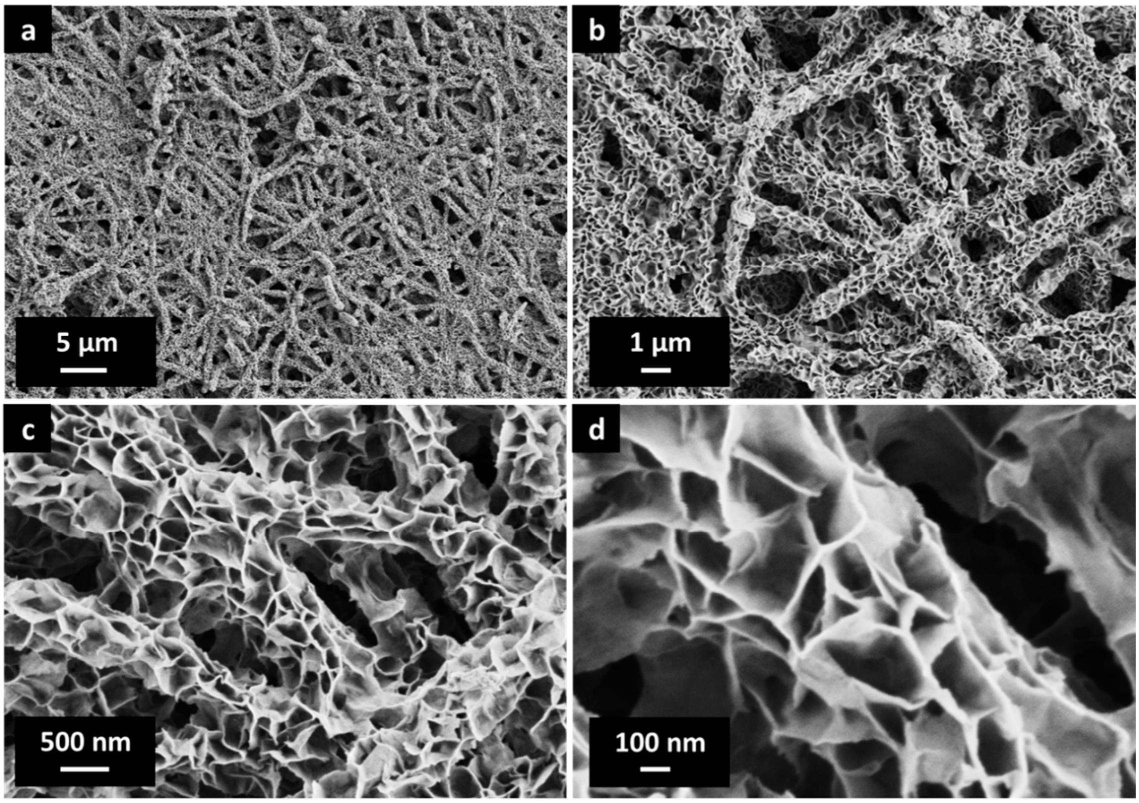

Figure S7: SEM images of pre-calcined AlNFs decorated by 7 wt% Zn and Cu (in a wt. ratio of 2.3 Zn/Cu) in the form of $Zn(NO_3)_2$ and $Cu(NO_3)_2$. (a)-(d) are magnifications of 5 k, 15 k, 50 k and 150 k, respectively.

Table S3 ICP and XRF results in wt%. Wt. % is defined as Me/(Al_2O_3+MeO), when Me can be Zn, Cu or both. Deviation superior to 1% from expected values are marked in orange.

			Measured and expected value [wt%]						Deviation from expected value [wt%]			
Catalyst Ref.		Metal loading of electrospun alumina nanofibers [wt%]	Cu			Zn			Cu		Zn	
			Measured		Expected	Measured		Expected	XRF vs expected	ICP vs expected	XRF vs expected	ICP vs expected
			XRF	ICP		ICP	XRF					
AlNFs-Zn catalysts	AlNFs-Zn-1.5	1.5% Zn	0.1	0.0	0.0	1.4	1.4	1.5	0.1	0.0	-0.1	-0.1
	AlNFs-Zn-3	3% Zn	0.1	0.0	0.0	2.9	3.1	2.9	0.1	0.0	0.0	0.2
	AlNFs-Zn-7	7% Zn	0.1	0.0	0.0	7.6	7.0	6.4	0.1	0.0	1.2	0.6
	AlNFs-Zn-10	10% Zn	0.1	0.0	0.0	13.4	9.4	8.9	0.1	0.0	4.5	0.5
AlNFs-Cu catalysts	AlNFs-Cu-1.5	1.5% Cu	1.8	1.4	1.5	0.1	0.0	0.0	0.3	-0.1	0.1	0.0
	AlNFs-Cu-3	3% Cu	3.4	2.6	2.9	0.1	0.0	0.0	0.6	-0.3	0.1	0.0
	AlNFs-Cu-7	7% Cu	7.7	5.6	6.4	0.1	0.0	0.0	1.3	-0.9	0.1	0.0
	AlNFs-Cu-10	10% Cu	12.1	9.2	8.9	0.0	0.0	0.0	3.2	0.3	0.0	0.0
AlNFs-CuZn catalysts	AlNFs-1Cu2.3Zn-1.5	1.5% Cu:Zn=1:2.3	0.5	0.3	0.4	1.2	1.0	1.0	0.0	-0.2	0.1	-0.1
	AlNFs-1Cu2.3Zn-3	3% Cu:Zn=1:2.3	0.9	0.7	0.9	1.9	1.8	2.0	0.0	-0.2	-0.1	-0.2
	AlNFs-1Cu2.3Zn-7	7% Cu:Zn=1:2.3	2.4	1.9	2.0	4.5	4.0	4.5	0.5	0.0	0.0	-0.5
	AlNFs-1Cu2.3Zn-10	10% Cu:Zn=1:2.3	3.3	2.4	2.7	6.3	5.1	6.2	0.6	-0.3	0.1	-1.1

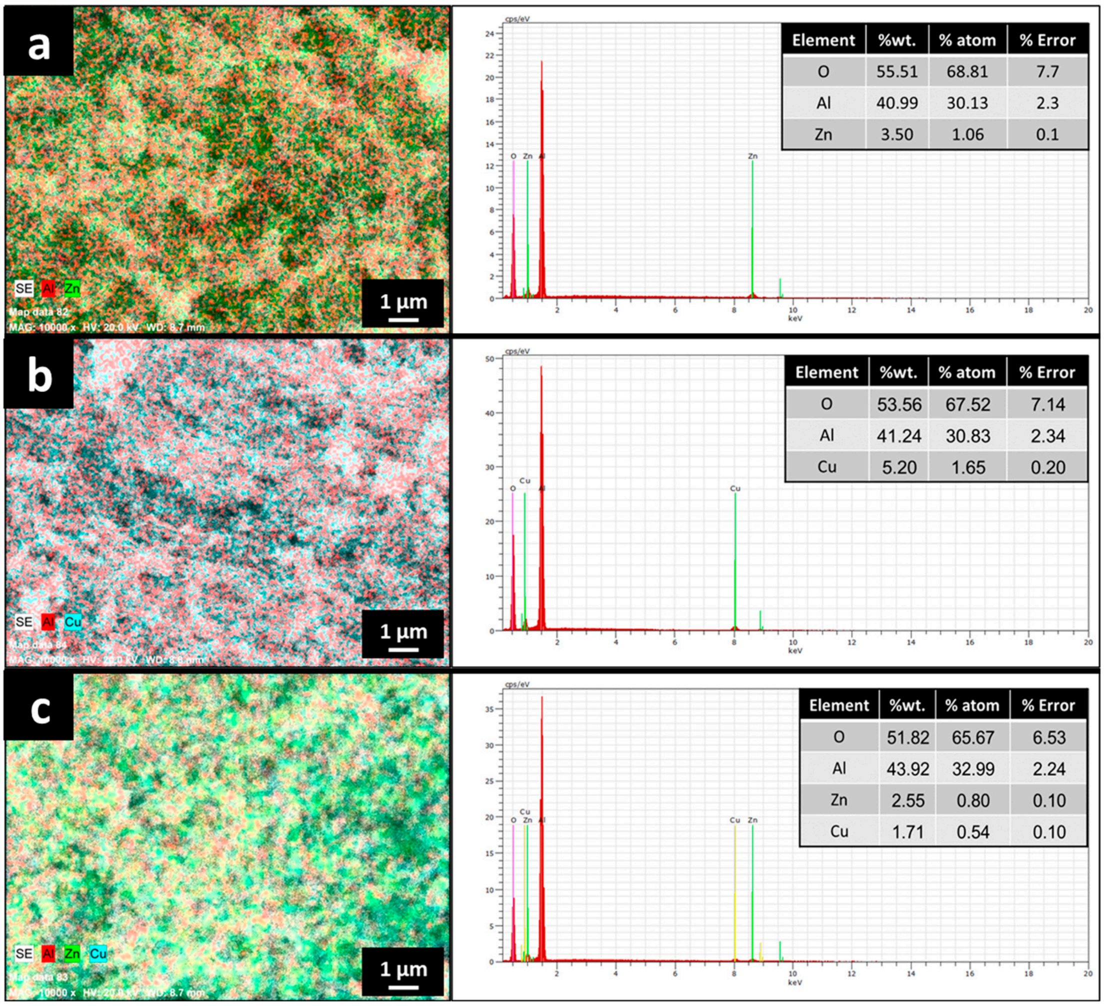

Figure S8: SEM-EDS results of decorated alumina nanofibers: (a) AlNFs-Zn-7 – alumina nanofiber decorated with 7 wt% of Zn (in form of ZnO); (b) AlNFs-Cu-7 – alumina nanofiber decorated with 7 wt% of Cu (in form of CuO); (b) AlNFs-1Cu2.3Zn-7 – alumina nanofiber decorated with 7 wt% of Cu and Zn (in form of CuO and ZnO) in a wt. ratio 1:2.3

Table S4: particles' sizes of AlNFs-1Cu2.3Zn-10 alumina nanofibers decorated with 10 wt% of Cu and Zn in a wt. ratio 1:2.3 as calculated by Scherrer equation.

Al-O NFs decorated by 10 wt% Zn		Al-O NFs decorated by 10 wt% Cu	
**2θ (°)**	**grains size (nm)**	**2θ (°)**	**grains size (nm)**
Zinc aluminum oxide (Al_2O_4Zn) gahnite phase		Copper aluminum oxide (Al_2CuO_4) spinel phase	
19.0	2.1	18.9	2.5
31.6	2.2	31.9	1.6
37.3	4.1	37.7	3.2
45.6	2.4	45.5	1.8
60.5	1.8	60.2	1.6
66.6	4.2	66.8	3.2
α-alumina (Al_2O_3) phase		α-alumina (Al_2O_3) phase	
25.6	52.3	25.6	75.7
26.3	24.5	26.2	9.8
35.2	45.0	35.2	47.1
----	---	37.8	62.5
43.4	51.7	43.4	46.8
52.6	54.5	52.6	45.3
57.5	59.3	57.5	50.5
66.5	22.7	66.5	31.7
68.2	50.7	68.2	31.2

CO_2 conversions [%]

	Pressure [bar]	10				30				50			
	Temperature [°C]	225	250	275	300	225	250	275	300	225	250	275	300
AlHFs-Zn catalysts	AlHFs-Zn-1.5	1,0	1,2	1,7	2,3	0,3	0,5	1,5	4,9	2,3	2,9	4,0	7,7
	AlHFs-Zn-3	0,4	1,2	2,0	3,5	0,6	1,0	2,1	6,3	2,0	3,4	5,1	9,8
	AlHFs-Zn-7	1,2	1,0	2,1	5,1	0,5	1,0	3,4	8,3	2,9	4,1	6,4	10,6
	AlHFs-Zn-10	0,4	0,5	1,7	3,6	1,1	0,8	2,4	6,4	3,1	3,6	5,1	6,8
AlHFs-Cu catalysts	AlHFs-Cu-1.5	1,8	3,8	5,7	8,4	1,2	2,9	5,4	9,6	3,0	4,7	6,6	5,0
	AlHFs-Cu-3	2,3	3,3	6,5	10,6	2,0	3,8	8,4	14,7	3,5	6,4	10,2	12,8
	AlHFs-Cu-7	4,5	10,5	18,6	25,3	4,8	11,1	21,2	27,7	6,6	14,4	23,3	27,5
	AlHFs-Cu-10	3,5	11,5	18,1	24,9	6,1	13,3	23,1	28,3	6,1	13,9	24,1	28,2
AlHFs-CuZn catalysts	AlHFs-1Cu2.3Zn-1.5	1,3	0,6	1,1	2,0	0,0	0,5	1,1	3,4	3,1	3,1	4,0	4,9
	AlHFs-1Cu2.3Zn-3	1,4	2,7	5,9	8,6	0,5	2,7	5,9	11,5	2,4	4,5	7,2	10,8
	AlHFs-1Cu2.3Zn-7	2,6	4,5	9,2	16,0	2,0	5,4	11,9	20,9	4,4	8,3	14,3	18,7
	AlHFs-1Cu2.3Zn-10	2,1	5,1	10,6	17,5	1,7	5,0	11,9	21,6	4,0	7,7	14,3	20,1

Table S5 CO_2 conversions for all catalysts at pressures of 10, 30, 50 bar and temperatures of 225, 250, 275, 300 °C.

Methanol Yield [%]

	Pressure [bar] =>	10				30				50			
	Temperature [°C] =>	225	250	275	300	225	250	275	300	225	250	275	300
AlHFs-Zn catalysts	AlHFs-Zn-1.5	0,0	0,0	0,0	0,0	0,0	0,1	0,0	0,0	0,1	0,0	0,0	0,1
	AlHFs-Zn-3	0,0	0,0	0,0	0,0	0,1	0,0	0,0	0,0	0,0	0,0	0,0	0,4
	AlHFs-Zn-7	0,0	0,0	0,0	0,1	0,0	0,0	0,3	0,5	0,0	0,0	0,2	0,6
	AlHFs-Zn-10	0,0	0,0	0,0	0,0	0,1	0,0	0,4	0,5	0,1	0,0	0,2	0,9
AlHFs-Cu catalysts	AlHFs-Cu-1.5	0,0	0,0	0,3	0,2	0,1	0,2	0,7	0,6	0,2	0,2	0,4	0,0
	AlHFs-Cu-3	0,0	0,1	0,4	0,4	0,3	0,5	1,1	1,2	0,3	0,8	1,1	1,9
	AlHFs-Cu-7	0,0	0,6	1,1	0,8	1,3	2,3	3,8	3,5	2,2	3,3	5,0	6,3
	AlHFs-Cu-10	0,7	1,1	1,4	0,6	1,8	2,9	3,9	3,8	3,0	4,5	6,1	7,0
AlHFs-CuZn catalysts	AlHFs-1Cu2.3Zn-1.5	0,0	0,0	0,0	0,0	0,1	0,0	0,0	0,0	0,0	0,0	0,0	0,1
	AlHFs-1Cu2.3Zn-3	0,0	0,0	0,3	0,3	0,1	0,3	0,8	0,8	0,2	0,3	0,5	0,4
	AlHFs-1Cu2.3Zn-7	0,1	0,3	0,7	0,8	0,6	1,1	2,1	2,3	0,9	1,4	2,4	3,1
	AlHFs-1Cu2.3Zn-10	0,0	0,1	0,7	0,8	0,7	1,1	2,1	2,5	1,0	1,6	2,7	3,4

Table S6 Methanol yield for all catalysts at pressures of 10, 30, 50 bar and temperatures of 225, 250, 275, 300 °C.

DME Yield [%]

	Pressure [bar] =>	10				30				50			
	Temperature [°C] =>	225	250	275	300	225	250	275	300	225	250	275	300
AlHFs-Zn catalysts	AlHFs-Zn-1.5	0,0	0,0	0,0	0,1	0,0	0,0	0,1	0,1	0,0	0,1	0,1	0,0
	AlHFs-Zn-3	0,0	0,1	0,1	0,1	0,0	0,0	0,1	0,2	0,0	0,1	0,2	0,2
	AlHFs-Zn-7	0,0	0,1	0,1	0,2	0,0	0,1	0,3	0,4	0,1	0,1	0,2	0,5
	AlHFs-Zn-10	0,0	0,0	0,1	0,1	0,0	0,0	0,2	0,4	0,0	0,1	0,2	0,6
AlHFs-Cu catalysts	AlHFs-Cu-1.5	0,2	0,3	0,2	0,1	0,6	0,5	0,7	0,4	0,3	0,6	0,3	0,1
	AlHFs-Cu-3	0,3	0,6	0,6	0,3	0,9	1,3	1,3	0,8	0,7	1,5	1,8	1,2
	AlHFs-Cu-7	1,0	1,2	1,1	0,3	2,1	3,0	3,4	3,0	2,0	3,2	4,4	6,0
	AlHFs-Cu-10	1,2	1,4	1,0	0,2	2,2	2,9	3,6	3,3	2,2	3,2	4,9	7,0
AlHFs-CuZn catalysts	AlHFs-1Cu2.3Zn-1.5	0,0	0,0	0,0	0,0	0,0	0,0	0,0	0,1	0,0	0,0	0,0	0,0
	AlHFs-1Cu2.3Zn-3	0,2	0,3	0,3	0,2	0,5	0,7	0,8	0,5	0,4	0,6	0,7	0,1
	AlHFs-1Cu2.3Zn-7	0,2	0,8	0,9	0,6	1,1	1,9	2,3	2,2	0,9	2,2	3,1	2,9
	AlHFs-1Cu2.3Zn-10	0,2	0,3	0,8	0,5	0,7	1,4	2,2	2,2	0,7	2,2	3,1	3,2

Table S7 DME yield for all catalysts at pressures of 10, 30, 50 bar and temperatures of 225, 250, 275, 300 °C.

CO Yield [%]

	Pressure [bar] =>	10				30				50			
	Temperature [°C] =>	225	250	275	300	225	250	275	300	225	250	275	300
AlHFs-Zn catalysts	AlHFs-Zn-1.5	0,0	0,0	1,3	2,6	0,0	0,4	2,0	4,2	0,0	1,0	2,4	6,3
	AlHFs-Zn-3	0,0	0,2	1,8	3,3	0,0	1,0	2,4	5,1	0,0	1,3	3,0	6,6
	AlHFs-Zn-7	0,0	0,2	2,6	4,5	0,0	1,3	3,2	6,2	0,0	1,3	3,5	8,2
	AlHFs-Zn-10	0,0	0,0	1,6	3,2	0,0	1,1	2,5	5,0	0,0	1,2	2,8	5,2
AlHFs-Cu catalysts	AlHFs-Cu-1.5	0,4	2,1	4,2	7,1	1,1	2,5	4,8	7,3	0,2	2,1	3,6	4,4
	AlHFs-Cu-3	1,2	2,7	5,1	8,5	1,3	2,9	6,0	10,2	1,2	3,5	6,1	8,0
	AlHFs-Cu-7	2,6	7,6	14,1	20,3	2,4	7,3	14,2	19,3	2,4	6,9	13,6	17,1
	AlHFs-Cu-10	2,8	7,1	14,1	20,4	3,1	8,3	15,4	19,2	3,1	8,7	14,1	16,9
AlHFs-CuZn catalysts	AlHFs-1Cu2.3Zn-1.5	0,0	0,0	0,6	2,0	0,0	0,2	1,6	3,8	0,0	0,8	2,2	5,3
	AlHFs-1Cu2.3Zn-3	0,3	2,4	4,1	7,1	0,3	2,4	5,0	8,8	1,0	2,1	4,6	8,2
	AlHFs-1Cu2.3Zn-7	1,0	3,2	7,2	12,0	1,4	4,1	8,6	15,0	1,6	3,6	8,1	12,7
	AlHFs-1Cu2.3Zn-10	1,1	3,5	7,3	12,9	1,3	3,4	8,1	15,8	1,2	3,6	8,5	14,2

Table S8 CO yield for all catalysts at pressures of 10, 30, 50 bar and temperatures of 225, 250, 275, 300 °C.

CH4 Yield [%]

	Pressure [bar] =>	10				30				50			
	Temperature [°C] =>	225	250	275	300	225	250	275	300	225	250	275	300
AlHFs-Zn catalysts	AlHFs-Zn-1.5	0,0	0,0	0,0	0,0	0,0	0,0	0,0	0,0	0,0	0,0	0,0	0,0
	AlHFs-Zn-3	0,0	0,0	0,0	0,0	0,0	0,0	0,0	0,0	0,0	0,0	0,0	0,0
	AlHFs-Zn-7	0,0	0,0	0,0	0,0	0,0	0,0	0,0	0,0	0,0	0,0	0,0	0,0
	AlHFs-Zn-10	0,0	0,0	0,0	0,0	0,0	0,0	0,0	0,0	0,0	0,0	0,0	0,0
AlHFs-Cu catalysts	AlHFs-Cu-1.5	0,0	0,1	0,1	0,2	0,0	0,1	0,2	0,3	0,0	0,1	0,2	0,3
	AlHFs-Cu-3	0,0	0,1	0,1	0,3	0,0	0,1	0,2	0,4	0,0	0,1	0,2	1,5
	AlHFs-Cu-7	0,0	0,0	0,1	0,2	0,0	0,1	0,3	0,7	0,1	0,2	0,4	0,9
	AlHFs-Cu-10	0,0	0,1	0,2	0,3	0,1	0,1	0,3	0,7	0,1	0,2	0,4	1,0
AlHFs-CuZn catalysts	AlHFs-1Cu2.3Zn-1.5	0,0	0,0	0,0	0,0	0,0	0,0	0,0	0,0	0,0	0,0	0,0	0,0
	AlHFs-1Cu2.3Zn-3	0,0	0,1	0,1	0,2	0,0	0,1	0,2	0,4	0,0	0,1	0,2	0,3
	AlHFs-1Cu2.3Zn-7	0,0	0,0	0,1	0,1	0,0	0,0	0,1	0,3	0,0	0,1	0,2	0,4
	AlHFs-1Cu2.3Zn-10	0,0	0,0	0,1	0,1	0,0	0,0	0,1	0,3	0,0	0,1	0,2	0,5

Table S9 CH_4 yield for all catalysts at pressures of 10, 30, 50 bar and temperatures of 225, 250, 275, 300 °C.

MeOH Production [g_{MeOH}/h/kg_{Cu}]

	Pressure [bar] =>	10				30				50			
	Temperature [°C] =>	225	250	275	300	225	250	275	300	225	250	275	300
AlHFs-Cu catalysts	AlHFs-Cu-1.5	11.6	0.0	119.0	102.5	57.1	107.9	308.7	272.4	75.6	69.8	171.1	18.3
	AlHFs-Cu-3	5.9	21.3	88.1	95.1	67.1	107.4	244.1	262.5	71.1	172.5	261.3	462.0
	AlHFs-Cu-7	0.0	60.0	113.6	80.1	130.1	230.1	385.3	352.3	220.5	336.8	513.3	686.1
	AlHFs-Cu-10	54.1	92.0	113.4	53.1	146.8	235.3	316.7	311.6	239.1	356.2	485.6	553.3
AlHFs-CuZn	AlHFs-1Cu2.3Zn-1.5	0.0	0.0	0.0	0.0	167.5	32.0	0.0	0.0	35.0	54.5	0.0	99.8
	AlHFs-1Cu2.3Zn-3	22.0	0.0	201.5	254.5	105.0	211.0	559.1	558.7	175.3	239.6	397.5	286.5
	AlHFs-1Cu2.3Zn-7	21.5	87.8	239.3	271.6	213.0	351.0	706.6	775.7	296.5	484.0	799.7	1106.3
	AlHFs-1Cu2.3Zn-10	0.0	35.2	168.2	198.4	169.3	254.7	502.4	590.7	241.6	384.0	657.5	894.3

Table S10 Space time yield of methanol per gram of Cu for AlNFs-Cu and AlNFs-1Cu2.3Zn catalysts at pressures of 10, 30, 50 bar and temperatures of 225, 250, 275, 300 °C.

DME Production [g_{DME}/h/kg_{Cu}]

	Pressure [bar] =>	10				30				50			
	Temperature [°C] =>	225	250	275	300	225	250	275	300	225	250	275	300
AlHFs-Cu catalysts	AlHFs-Cu-1.5	63.4	103.2	75.6	44.0	174.5	173.7	225.7	140.8	105.7	178.3	104.9	26.2
	AlHFs-Cu-3	45.7	96.0	88.6	44.9	152.8	204.1	216.4	132.2	107.4	241.4	288.8	217.0
	AlHFs-Cu-7	71.4	88.5	75.2	19.6	153.6	213.6	245.9	219.2	150.3	234.7	322.1	469.2
	AlHFs-Cu-10	71.3	81.8	61.6	13.0	128.9	170.6	211.3	195.4	122.7	184.3	278.0	397.1
AlHFs-CuZn	AlHFs-1Cu2.3Zn-1.5	0.0	0.0	0.0	19.9	0.0	0.0	25.9	88.9	0.0	21.0	35.9	0.0
	AlHFs-1Cu2.3Zn-3	117.7	174.6	172.2	108.8	251.4	381.9	412.0	272.0	207.0	320.1	370.7	81.0
	AlHFs-1Cu2.3Zn-7	49.0	188.4	209.6	135.6	251.9	441.8	557.0	532.7	222.0	530.9	757.2	760.4
	AlHFs-1Cu2.3Zn-10	26.9	48.7	145.2	88.4	117.6	245.5	388.7	379.0	117.6	378.0	546.4	605.6

Table S11 Space time yield to DME per gram of Cu for AlNFs-Cu and AlNFs-1Cu2.3Zn catalysts at pressures of 10, 30, 50 bar and temperatures of 225, 250, 275, 300 °C.

Production of the Oxygenated products - MeOH and DME [$g_{MeOH+DME}$/h/kg_{Cu}]

	Pressure [bar] =>	10				30				50			
	Temperature [°C] =>	225	250	275	300	225	250	275	300	225	250	275	300
AlHFs-Cu catalysts	AlHFs-Cu-1.5	75.1	103.2	194.6	146.6	231.7	281.6	534.4	413.1	181.3	248.1	276.1	44.5
	AlHFs-Cu-3	51.6	117.3	176.7	140.0	219.9	311.5	460.5	394.7	178.5	413.9	550.1	679.0
	AlHFs-Cu-7	71.4	148.5	188.8	99.8	283.7	443.6	631.2	571.5	370.8	571.5	835.4	1155.3
	AlHFs-Cu-10	125.4	173.8	175.0	66.1	275.7	406.0	528.0	507.0	361.8	540.4	763.6	950.4
AlHFs-CuZn	AlHFs-1Cu2.3Zn-1.5	0.0	0.0	0.0	19.9	167.5	32.0	25.9	88.9	35.0	75.5	35.9	99.8
	AlHFs-1Cu2.3Zn-3	139.7	174.6	373.8	363.3	356.4	592.9	971.1	830.7	382.3	559.8	768.2	367.5
	AlHFs-1Cu2.3Zn-7	70.5	276.2	448.9	407.2	464.9	792.7	1263.6	1308.3	518.5	1014.9	1556.9	1866.7
	AlHFs-1Cu2.3Zn-10	26.9	83.9	313.4	286.8	286.9	500.2	891.1	969.6	359.1	762.1	1203.8	1499.9

Table S12 Space time yield to Methanol and DME per gram of Cu for AlNFs-Cu and AlNFs-1Cu2.3Zn catalysts at pressures of 10,30,50 bar and temperatures of 225, 250, 275, 300 °C.

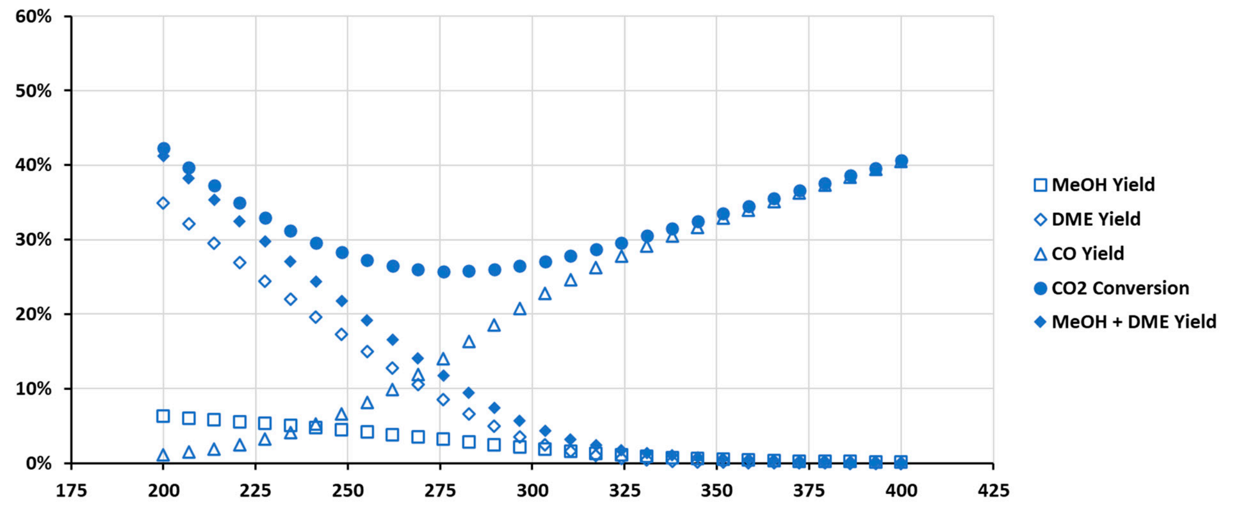

Figure S9: Effect of temperature on CO_2 conversion and yields to Methanol, Dimethyl ether and CO at equilibrium at 30 bar pressure and H_2/CO_2=4 ratio.

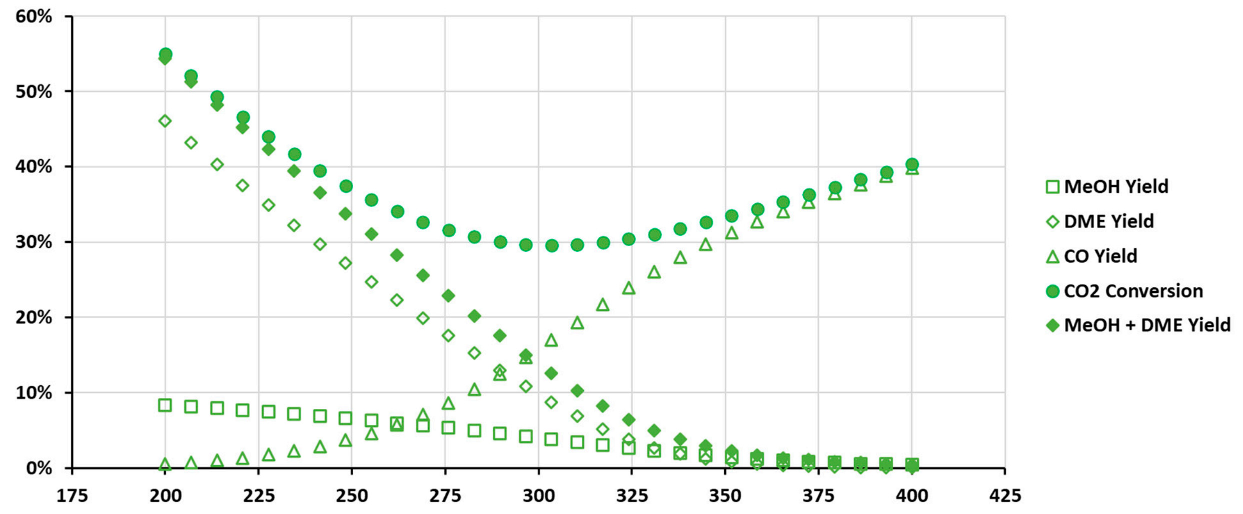

Figure S10: Effect of temperature on CO_2 conversion and yields to Methanol, Dimethyl ether and CO at equilibrium at 50 bar pressure and H_2/CO_2=4 ratio.

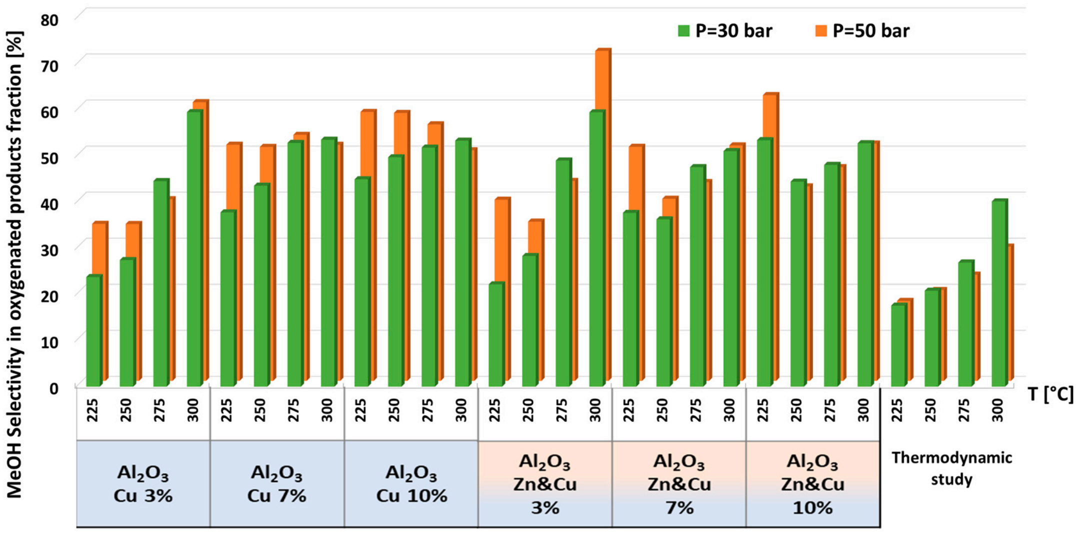

Figure S11. Selectivity to methanol in oxygenated products (MeOH and DME) fraction for some AlNFs-Cu and AlNFs-1Cu2.3Zn catalysts at pressures of 30 and 50 bar and temperatures of 225, 250, 275, 300 °C.

References:

1. Wang, L.; Etim, U.J.; Zhang, C.; Amirav, L.; Zhong, Z. CO2 Activation and Hydrogenation on Cu-ZnO/Al2O3 Nanorod Catalysts: An In Situ FTIR Study. *Nanomaterials* **2022**, *12*, 1–15, doi:10.3390/nano12152527.

2. Navarro-Jaén, S.; Virginie, M.; Thuriot-Roukos, J.; Wojcieszak, R.; Khodakov, A.Y. Structure–Performance Correlations in the Hybrid Oxide-Supported Copper–Zinc SAPO-34 Catalysts for Direct Synthesis of Dimethyl Ether from CO2. *J. Mater. Sci.* **2022**, *57*, 3268–3279, doi:10.1007/s10853-022-06890-w.

3. Lam, E.; Corral-Pérez, J.J.; Larmier, K.; Noh, G.; Wolf, P.; Comas-Vives, A.; Urakawa, A.; Copéret, C. CO2 Hydrogenation on Cu/Al2O3: Role of the Metal/Support Interface in Driving Activity and Selectivity of a Bifunctional Catalyst. *Angew. Chemie - Int. Ed.* **2019**, *58*, 13989–13996, doi:10.1002/anie.201908060.

4. Navarro-Jaén, S.; Virginie, M.; Morin, J.C.; Thuriot-Roukos, J.; Wojcieszak, R.; Khodakov, A.Y. Hybrid Monometallic and Bimetallic Copper-Palladium Zeolite Catalysts for Direct Synthesis of Dimethyl Ether from CO2. *New J. Chem.* **2022**, *46*, 3889–3900, doi:10.1039/d1nj05734k.

5. Carvalho, D.F.; Almeida, G.C.; Monteiro, R.S.; Mota, C.J.A. Hydrogenation of CO2to Methanol and Dimethyl Ether over a Bifunctional Cu·ZnO Catalyst Impregnated on Modified γ-Alumina. *Energy and Fuels* **2020**, *34*, 7269–7274, doi:10.1021/acs.energyfuels.0c00680.
